# Changes in Proteome of Fibroblasts Isolated from Psoriatic Skin Lesions

**DOI:** 10.3390/ijms21155363

**Published:** 2020-07-28

**Authors:** Agnieszka Gęgotek, Pedro Domingues, Adam Wroński, Elżbieta Skrzydlewska

**Affiliations:** 1 Department of Analytical Chemistry, Medical University of Bialystok, Mickiewicza 2D, 15-222 Bialystok, Poland; agnieszka.gegotek@umb.edu.pl; 2 Mass Spectrometry Centre, LAQV REQUIMTE, Department of Chemistry, University of Aveiro, 3810-193 Aveiro, Portugal; p.domingues@ua.pt; 3 Dermatological Specialized Center “DERMAL” NZOZ in Bialystok, 15-453 Bialystok, Poland; adam.wronski@dermal.pl

**Keywords:** psoriasis, skin fibroblasts, proteomic profile, inflammation, oxidative conditions, intracellular signal transduction

## Abstract

The dermal fibroblasts are in constant contact with the cells of the immune system and skin epidermis. Therefore, they are essential for the development of lesions in psoriasis. The aim of this study was to assess the changes in the proteomic profile of fibroblasts in the dermis of psoriasis patients, and to discuss the most significant changes and their potential consequences. The proteomic results indicate that fibroblast dysfunction arises from the upregulation of proinflammatory factors and antioxidant proteins, as well as those involved in signal transduction and participating in proteolytic processes. Moreover, downregulated proteins in psoriatic fibroblasts are mainly responsible for the transcription/translation processes, glycolysis/ adenosine triphosphate synthesis and structural molecules. These changes can directly affect intercellular signaling and promote the hyperproliferation of epidermal cells. A better understanding of the metabolic effects of the proteomic changes observed could guide the development of new pharmacotherapies for psoriasis.

## 1. Introduction

Psoriasis is a chronic disease that occurs with increasing frequency in developed countries. In European countries and the United States, the prevalence of psoriasis can reach 3% [1,2]. Psoriasis occurs mainly due to a dysfunction of the immune system, and its development might be associated with other diseases, including arthritis, metabolic syndrome, heart disease, polycystic ovarian syndrome, chronic obstructive pulmonary disease and even cancer [3]. However, the main symptom of the disease is excessive skin exfoliation [4]. As a result, psoriasis also affects the psychophysical health of patients by lowering their self-esteem and disrupting their social behavior. Depressive symptoms and sleep disturbances are also common in psoriatic patients [5,6].

The pathogenesis of psoriasis is the result of impaired immunity, and has a genetic component linked to the immune genes and their encoded pathways, as well as to environmental factors such as drugs, smoking, diet, alcohol and mental stress [7]. Regardless of the particular mechanisms involved, psoriasis develops due to the chronic activation of the cells of the peripheral immune system, resulting in the increased proliferation and differentiation of skin cells [8,9]. Significant changes occur in the epidermis, where the accelerated cell cycle of keratinocytes results in intensified keratinization and the formation of cutaneous psoriatic lesions. Epidermal keratinocytes are stimulated to proliferate by signaling molecules, primarily released by lymphocytes. This process has been well examined and described previously [8].

Undoubtedly, the release of signaling molecules that can reach and interact with the epidermis will also have an impact on cells that build the other layers of the skin, including dermal fibroblasts. Under physiological conditions, skin fibroblasts are primarily responsible for the production of collagen and other intercellular matrix substances present in the dermis, which are intimately linked to the condition and function of the skin [10]. However, the metabolic activity of fibroblasts in psoriatic skin has not been extensively studied in recent years, compared to keratinocytes, which have been the subject of extensive research [11,12,13,14,15,16,17,18].

Oxidative stress is a characteristic of the tissue of patients with psoriasis [19]. Currently, it is known that oxidative stress in dermal fibroblasts is higher in scaly skin than in unchanged tissue [20]. It is important to note that the increase in oxidative stress and the decrease in the total antioxidant capacity of dermal fibroblasts are even greater than in the keratinocytes isolated from the same skin biopsy [20]. Under such conditions, the molecules present in intracellular fibroblasts may undergo oxidative modifications, which can trigger an increase in oxidative lipid metabolism [21]. As a result, there is an increase in lipid peroxidation products, including reactive α, β-unsaturated aldehydes and isoprostanes [22]. Additionally, the increase in the enzymatic lipid metabolism of psoriatic fibroblasts promotes the production of bioactive mediators, including eicosanoids, sphingolipids and ceramides. These mediators are involved in skin biology, inflammation and immunity, and even cell apoptosis [23,24].

Increased levels of electrophilic molecules, mainly reactive oxygen species (ROS), as well as reactive aldehydes, especially 4-hydroxynenenal (4-HNE) and malondialdehyde (MDA), can also lead to modifications of proteins in patients with psoriasis. These modifications have been observed in lymphocytes and keratinocytes, and included the formation of protein adducts with lipid peroxidation products [17,25] and a significant increase in protein carbonylation in skin fibroblasts [20]. The presence of these protein modifications in psoriatic fibroblasts also leads to the activation of redox-sensitive signaling pathways, including those that depend on the mitogen-activated protein kinases (mitogen-activated protein kinase (MAPK), p38, extracellular signal-regulated kinase (ERK) and c-Jun N-terminal kinase (JNK)) [21], as well as protein kinase C (PKC) [26]. Consistently, PKC in the cell membranes of psoriatic fibroblasts is significantly activated, which could make these cells very sensitive in response to hormones or growth factors [26]. Moreover, psoriatic fibroblasts, unlike unmodified dermal cells, have been shown to stimulate the proliferation of keratinocytes after receiving activation signals [27]. An example of such action in psoriatic fibroblasts stimulated by inflammatory cytokines is the observation that increased expression of the insulin-like growth factor-I (IGF-I) significantly promotes the proliferation of keratinocytes [28]. Metabolic disturbances in psoriatic fibroblasts also cause increased expression of interleukin 8 (IL-8), resulting in the stimulation of neutrophils, monocytes and T lymphocytes, which migrate into the skin layers [29]. In addition, the changes observed following psoriatic epidermal exfoliation are linked to changes in the metabolism of fibroblasts, not only locally but also in regions distant from the exfoliation site. The expression of factors such as α5 integrin, fibronectin or keratinocyte growth factor (KGF) is high, in particular in non-lesional psoriatic skin fibroblasts [30]. In agreement with this, it is suggested that these factors play a crucial role in the pathogenesis of psoriasis by influencing the inflammation and hyperproliferation of keratinocytes.

The abundance of evidence highlighting the critical role of fibroblasts in the development of psoriasis lesions has led us to investigate in more detail the molecular mechanisms leading to the pathogenesis of the disease. To achieve this, we sought to determine the differences in the proteomic profiles of fibroblasts isolated from the dermis of psoriatic patients, compared to unmodified skin cells.

## 2. Results

**Figure 1:**
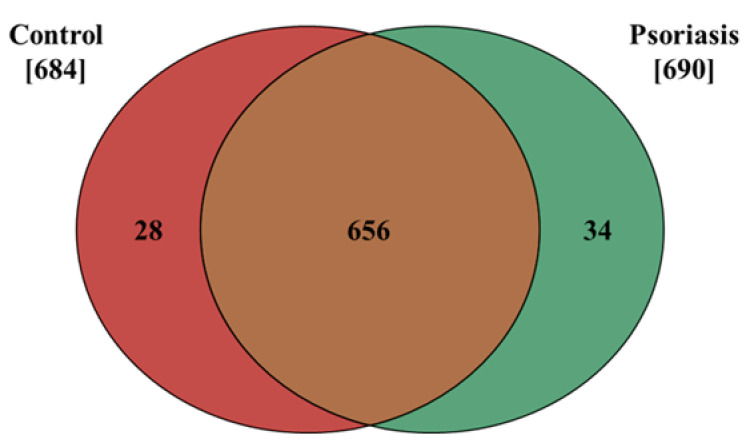
Venn diagram showing the number of proteins in fibroblasts isolated from the skin of psoriatic patients (*n* = 5) and healthy controls (*n* = 5). The names and ID of all the proteins identified are contained in the Supplementary Table S1.

The results presented in this study show that the proteome of fibroblasts isolated from the dermis of psoriatic patients has a different profile than that of control cells. The data obtained from our proteomic analysis allowed us to identify and semi-quantitatively determine the expressions of 718 proteins, 684 of which were found in control fibroblasts, and 690 in cells isolated from the skin of psoriatic patients ( Supplementary Table S1). The distribution of these proteins between the samples is shown on a Venn diagram (Figure 1).

**Figure 2:**
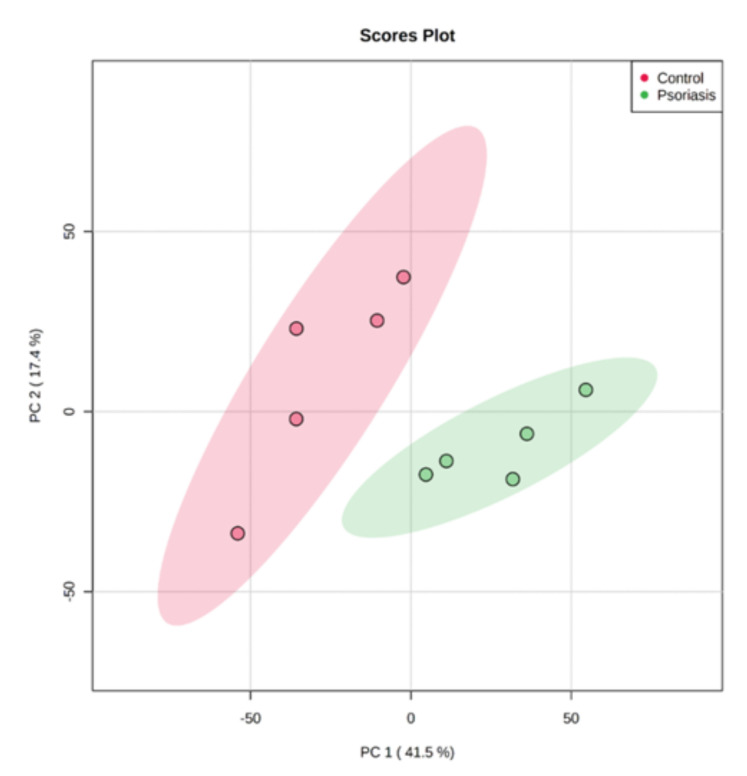
Principal component analysis (PCA) of the proteins in fibroblasts isolated from skin of psoriatic patients (*n* = 5) and healthy controls (*n* = 5).

**Figure 3:**
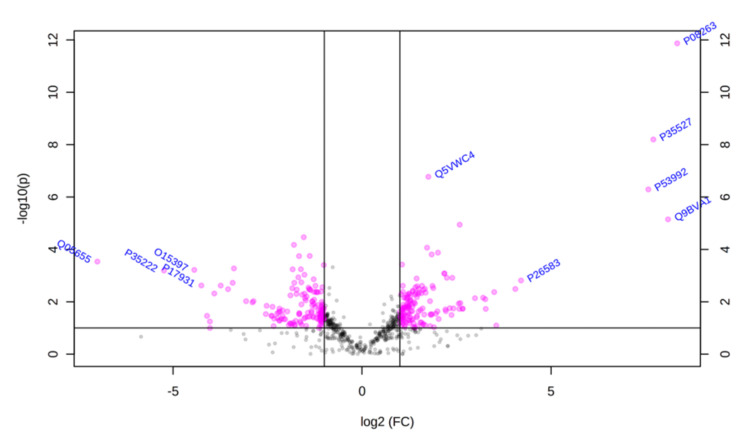
Volcano plot of fibroblasts proteins isolated from the skin of psoriatic patients (*n* = 5) and healthy controls (*n* = 5). Red dots indicate proteins of statistical significance among the groups tested. The *p-*values and the fold change (FC) for each protein are included in Supplementary Table S2.

Using principal component analysis (PCA), we found that changes in the proteomic profiles of skin fibroblast cells led to the clustering of the experimental groups (PC1—41.5%, PC2—17.4%). In the case of control fibroblasts, the samples clustered in the left quadrant, while the psoriatic fibroblasts clustered mainly in the lower right quadrant (Figure 2). Statistical analysis indicated that the expressions of 242 of the proteins identified were significantly different between the control fibroblasts and the psoriatic fibroblasts ( Supplementary Table S2). A volcano plot displaying differentially enriched proteins highlighted that the most significantly changed proteins in psoriatic fibroblasts were downregulated; β-catenin (P35222), importin-8 (O15397), protein kinase C (Q05655) and galectin-3 (P17931). The following, on the other hand, were upregulated: keratin (P35527), tubulin (Q9BVA1), 26S proteasome (Q5VWC4), protein transport protein Sec24C (P53992), glutathione S transferase 1 (P08263) and high mobility group protein B2 (P26583) (Figure 3).

**Figure 4:**
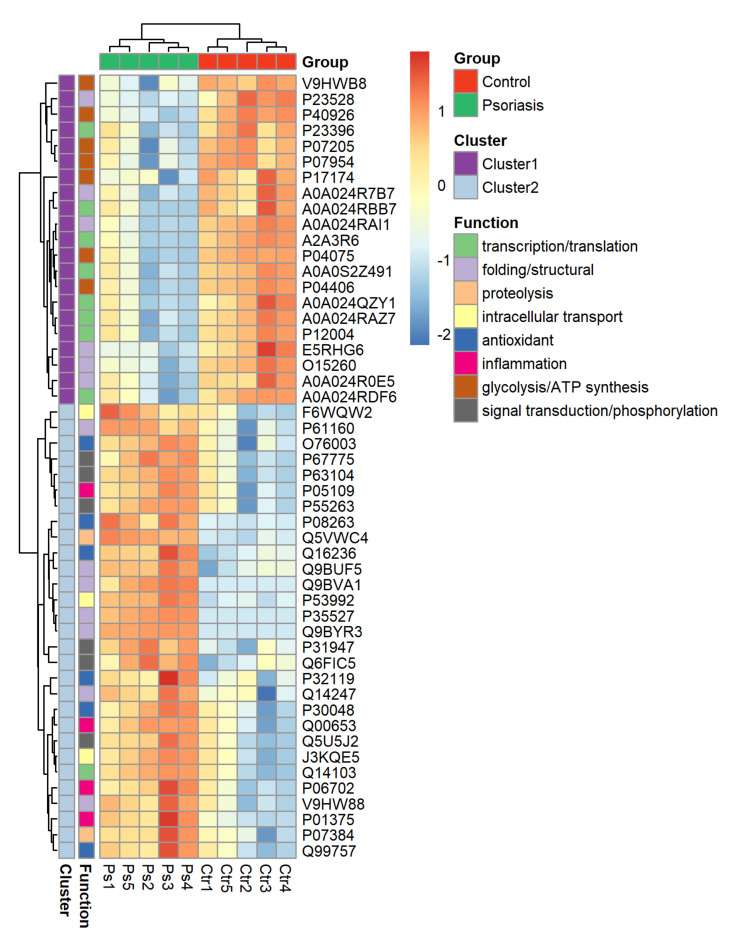
Heat map and clustering of the 50 most significantly changing proteins of fibroblasts isolated from the skin of psoriatic patients (*n* = 5) and healthy controls (*n* = 5).

**Figure 5:**
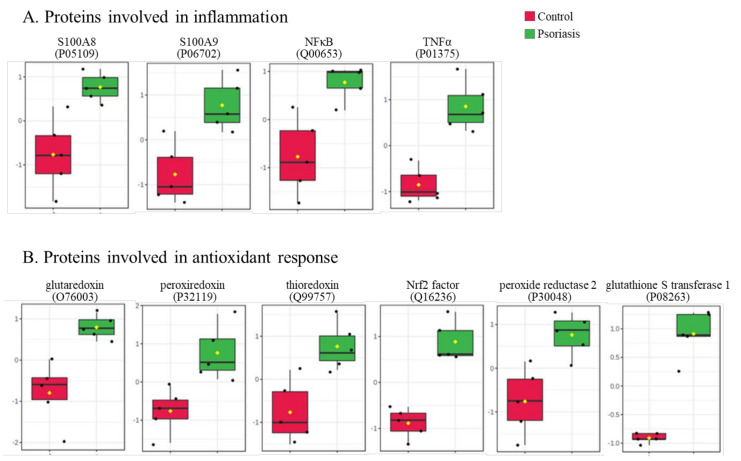
The level of significantly changed proteins involved in the antioxidant response (**A**) and inflammation (**B**) of fibroblasts isolated from skin of psoriatic patients (*n* = 5) and healthy controls (*n* = 5).

**Figure 6:**
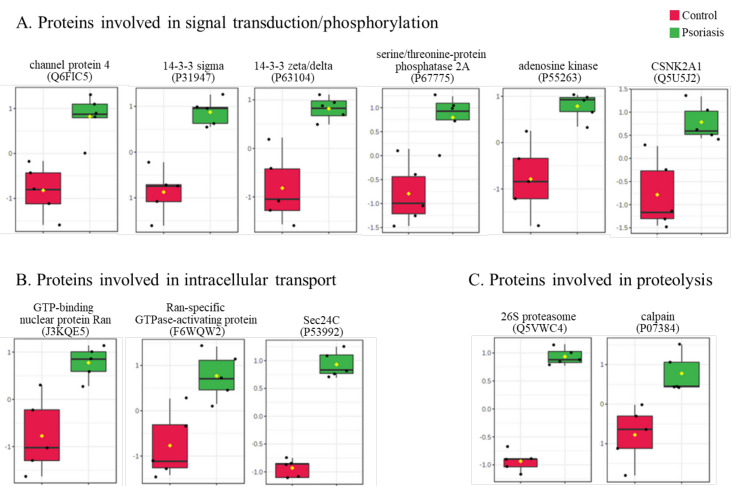
The level of significantly changed proteins involved in signal transduction/phosphorylation (**A**), intracellular transport (**B**) and proteolysis (**C**) of fibroblasts isolated from the skin of psoriatic patients (*n* = 5) and healthy controls (*n* = 5).

The clustering and functions of the 50 most significant proteins were visualized in a two-dimensional hierarchical clustering heat map (Figure 4). The analyzed proteins were divided into two clusters. Cluster 1 contained proteins downregulated in psoriatic fibroblasts compared to controls. These proteins were primarily involved in the transcription/translation processes, protein folding and glycolysis/ATP synthesis, as well as having structural functions. Cluster 2 contained proteins upregulated in psoriasis and had various different biological functions. Some of them were proinflammatory proteins, including NFκB (Q00653), TNFα (P01375) and S100A8/9 (P05109/P06702) (Figure 5A). Another group of proteins upregulated in psoriatic fibroblasts was those with antioxidant properties. These included thioredoxin (Q99757), peroxiredoxin (P32119), glutaredoxin (O76003), Nrf2 (Q16236), glutathione S transferase 1 (P08263) and thioredoxin-dependent peroxide reductase 2 (P30048) (Figure 5B). Finally, psoriatic fibroblasts were characterized by higher expressions of proteins involved in signal transduction (such as 14-3-3 proteins (P31947, P63104), kinases (P55263, P67775, Q5U5J2) and intracellular channel protein 4 (Q6FIC5)) (Figure 6A), intracellular transport (such as the Ran-specific GTPase-activating protein (F6WQW2), GTP-binding nuclear protein Ran (J3KQE5) and transport protein Sec24C (P53992)) (Figure 6B) and the proteins involved in the proteolytic processes (calpain (P07384) and 26S proteasome (Q5VWC4)) (Figure 6C).

## 3. Discussion

Psoriasis is an immune-mediated disease characterized by increased activation of lymphocytes. Once the lymphocytes have entered the skin, they cause the proliferation, maturation and desquamation of keratinocytes [25]. The resulting skin lesions disrupt the functioning of the epidermis and can lead to a reduction in the quality of life of those affected [5,31]. However, the development of psoriasis is not only linked to changes in the interaction between lymphocytes and keratinocytes; it also involves the activity of other cells of the immune system. For example, the actions of granulocytes [32,33] can significantly alter the profile of the signaling molecules in the plasma of psoriatic patients [34]. Such changes do not exclusively affect keratinocytes, and also have consequences for other skin-building cells, including dermal fibroblasts [24,30,35].

Fibroblasts, as the primary cells of the dermis, are responsible for the synthesis of proteoglycans, glycosaminoglycans and collagen, which are the main components of the extracellular matrix. These cells are essential for maintaining the appropriate thickness of the dermis when the exfoliated epidermis becomes too thin to fulfill a protective function. Therefore, increased collagen biosynthesis in these cells is observed in psoriatic skin [36,37]. Moreover, fibroblasts directly promote the proliferation of keratinocytes by generating and transporting growth factors [27].

These observations highlight the need for further research into how changes in the proteome of these cells could contribute to the development of psoriasis. To date, complex changes in the proteome of psoriatic patients have been identified in keratinocytes and biopsies of the whole skin, as well as in blood cells and plasma [35,38,39,40,41,42,43]. However, in fibroblasts from psoriatic patients, only changes in candidate proteins were analyzed [20,21,26,28,30,44]. Our study investigated changes in the proteome of psoriatic fibroblasts. In this report, we present the most significant modifications and discuss their potential consequences. The changes described include the upregulated proteins that are involved in inflammation, the antioxidant response, signal transduction and proteolytic processes, as well as the downregulated proteins mainly responsible for the transcription/translation processes, glycolysis/ATP synthesis and structural components.

Psoriatic skin exfoliation is intrinsically linked to the response of skin cells to proinflammatory factors generated by immune cells [45]. These agents activate cell membrane receptors leading to stimulation of the intracellular pro-inflammatory pathways. Consequently, the expression of NFκB is increased in dermal fibroblasts, but also in epidermal keratinocytes [46]. The action of NFκB not only leads to the expression of the genes responsible for the inflammatory reaction, but also stimulates other pro-inflammatory factors, including TNFα, which intensify pro-inflammatory signaling within the cell and between adjacent cells. In psoriatic plaques, the skin levels of TNFα are increased, which promotes infiltration by macrophages that also express TNFα [47,48,49]. As a result, keratinocytes are continuously stimulated to proliferate [50]. Until now, there has been no unambiguous data indicating the levels of these factors in psoriatic fibroblasts. However, the presented results allow us to suggest that the increased expression of NFκB and TNFα in fibroblasts can enhance intercellular proinflammatory signaling, thus contributing to the stimulation of keratinocytes proliferation.

Moreover, the increase in the level of pro-inflammatory factors in psoriatic fibroblasts is accompanied by an increased expression of the proteins participating in the proteolytic processes, such as 26S proteasome components and S100A8/A9. The ubiquitin–proteasome pathway activated during the development of psoriasis also plays a central role in the selective degradation of intracellular proteins, including those involved in the control of inflammatory processes [51]. The increase in the level of 26S proteasome subunits observed in this study could contribute to an increased degradation of IκB, which is a cytosolic inhibitor of NFκB [52,53]. As a result, NFκB, by improving the inflammatory response, promotes T cell responses in psoriasis [54]. In vitro and in vivo experiments have shown that 26S proteasome inhibitors inhibit cell proliferation and migration under inflammatory conditions [55], highlighting the potential for the inhibition of the proteasome as a treatment option for inflammatory disorders such as psoriasis [51]. Another protein with proteolytic activity associated with inflammation is a calcium-dependent neutral protease called calpain. The main role of calpain is the regulation of various fundamental cellular functions, such as the cell cycle and apoptosis, but it is also involved in the initiation of inflammation by the degradation of IκB and the activation of NFκB [56]. An increase in the level of calpain in psoriatic patients has already been identified in the skin tissue [57,58]. However, calpain also stimulates the migration of fibroblasts and myoblasts, which is necessary for the treatment of damaged skin [59].

Our data also show an increase in the level of S100A8/A9 proteins in psoriatic fibroblasts. Previous studies have determined that the source of S100A proteins in skin tissue are the activated phagocytes in inflammatory conditions associated with psoriasis lesions [60]. The main role of S100A8/A9 in the psoriatic epidermis is to activate the complement component 3 protein (C3) in keratinocytes. After activation, C3 is translocated to the dermis, where it stimulates immune cells to produce cytokines, interleukins and growth factors, thereby contributing to the development of psoriasis [61,62,63]. It is unknown whether psoriatic fibroblasts can synthesize S100A8/A9 or can only accumulate proteins when produced by other cells. However, our data, which show a significant increase in these proteins in dermal cells, suggest an additional role for fibroblasts in pro-inflammatory signaling, which leads to the hyperproliferation of keratinocytes in psoriasis.

Inflammatory diseases, such as psoriasis, are associated with pro-oxidative conditions, leading to oxidative stress [64,65]. In response, the level and activity of components of the antioxidant system increase in patients with psoriasis [66,67]. Our results confirm that in the fibroblasts of psoriasis patients, one of the main groups of significantly modified proteins is the proteins involved in the antioxidant response. These include the transcription factor Nrf2—a redox-sensitive protein responsible for the expression of cytoprotective proteins. Various investigations into psoriatic keratinocytes have observed changes in Nrf2 levels. One study found that a decrease in the levels of Nrf2 was associated with the development of psoriasis [68], while others observed an increased expression of Nrf2, which led to the elevated expression of keratins and promoted the proliferation of keratinocytes, leading to the pathogenesis of psoriasis [69,70]. The transcriptional activity of Nrf2 leads to the expression of genes coding for antioxidant enzymes, in particular thioredoxin-dependent peroxide reductase and glutathione S transferase 1 [71], the levels of which are increased in psoriatic fibroblasts. A previous study also indicated that the level of these enzymes is increased in fibroblasts under oxidative stress induced by UV, which is probably a defense mechanism against adverse conditions in the cell [72]. Moreover, the increased level of thioredoxin-dependent peroxide reductase is accompanied by a high level of thioredoxin, which is associated with the increased activity of this enzyme. Simultaneously, the levels of peroxiredoxin and glutaredoxin are increased. These proteins can reduce thiol groups in oxidized proteins and also control the peroxide levels induced by cytokines [73]. Previous reports confirm the increase in the mentioned parameters of the antioxidant system in skin biopsies of psoriatic patients [74]. Along with the previously published data, our findings indicate that fibroblasts from psoriasis patients are subject to high levels of oxidative stress, and these cells activate pathways to limit these oxidative conditions.

Signal transduction between cells involved in psoriatic lesion development is one of the fundamental elements to consider in designing effective treatments for psoriasis [75,76,77]. So far, the role of fibroblasts in this intercellular communication has not been described. In this study, we found that fibroblasts in psoriatic skin display the upregulation of 14-3-3 sigma (σ) and zeta/delta (ζ/δ) protein isoforms. Other studies show that 14-3-3 protein levels in psoriatic skin biopsies are changed in various ways, depending on the isoform; 14-3-3τ and σ are upregulated [78,79,80], while 14-3-3β and 14-3-3ζ are downregulated [81]. 14-3-3 is involved in the regulation of transcription and translation through its interaction with DNA/mRNA-binding proteins, such as tristetraprolin (TTP), which induces the destabilization and degradation of cytokine mRNA (including TNFα mRNA). After phosphorylation, TTP can bind to 14-3-3, which inhibits the mRNA-degrading capabilities of TTP. Therefore, in several skin diseases characterized by hyperproliferative keratinocytes, increased levels of 14-3-3 result in the overexpression of cytokines [78]. These changes are accompanied by the upregulation of kinases, as shown in this study and in previous work on a psoriatic skin model [82]. Conversely, in the case of DNA-damage or the overexpression of dysregulated genes, 14–3–3σ is upregulated by a p53-dependent pathway, and partially prevents cells from entering mitosis [83].

In psoriatic skin fibroblasts, the expression of the chloride intracellular channel protein 4 (ClIC4) is increased. The activity of this transmembrane protein is linked to angiogenesis and to the differentiation of keratinocytes [84]. In the case of fibroblasts, the overexpression of ClIC4 leads to the activation of the transforming growth factor-β1 (TGF-β1) and to conversion to myofibroblasts, which is a known feature of diseases characterized by the hyperproliferation of cells, including cancer [44,85]. Consistent with this finding, increased levels of TGF-β1 have been observed in the keratinocytes, plasma and lymphocytes of psoriatic patients [86,87,88]. Therefore, the increased ClIC4 level in fibroblasts from psoriatic patients suggests the role of these cells in enhancing the expression of TGF-β1 in the tissues of people affected by psoriasis.

Psoriatic fibroblasts are also characterized by the increased expression of proteins involved in intracellular transport. Intracellular transport is essential for cells with accelerated proliferation, as well as those that are constantly exposed to the signaling molecules released by cells of the immune system. At the top of the list of the most-changing expressed proteins are the Ran-specific GTPase-activating protein (RANBP1) and the GTP-binding nuclear protein Ran. The actions of these proteins lead to the selective activation of Ran [89], a protein involved in the transport of proteins across the nuclear membrane. Ran carries out nuclear transport by binding to importins or exportins, and thus participates in the activity of regulation of the transcription factor [89]. So far, the increase in the level of Ran and its activators has been identified in activated human lymphocytes T [90]. However, despite the importance of the Ran-related pathway in regulating the cell cycle, it has not been widely analyzed in samples from psoriatic patients. Moreover, the Sec24C protein is also upregulated in psoriatic fibroblasts. The activity of Sec24C facilitates the selection of proteins for transport to the cell nucleus, but is also involved in the transport of proteins to the endoplasmic reticulum [91]. Therefore, Sec24C is responsible for the proper biosynthesis, maturation and secretion of collagen [92], suggesting that its upregulation plays a specific role in the fibroblasts of regularly exfoliated psoriatic skin.

The fibroblasts of psoriatic skin lesions also contain a large group of proteins whose expression is reduced compared to healthy cells. The main proteins are β-catenin, importin-8 and galectin-3. All of these proteins are involved in cell–cell adhesion, which is an essential process in the formation of the skin layers [93,94,95]. Decreased levels of β-catenin have been found in the cytoplasm of keratinocytes in psoriatic skin [93]. β-catenin is responsible for the transmission of the contact inhibition signal, which causes the division of cells to stop. Therefore, its deficiency in psoriasis does not stop cell division. However, in psoriatic cells, β-catenin accumulates in the nucleus, where it can upregulate gene expression and influence cell growth [93]. Conversely, gene expression might also be dysregulated by a decrease in the level of importin-8. This protein is responsible for the transport of mature miRNAs from the cytoplasm to the nucleus, which causes gene silencing [94]. Cells deficient in importin-8 display uncontrolled gene expression [96] which, in the skin, can lead to the hyperproliferation of keratinocytes and the formation of psoriatic lesions.

Another downregulated protein in psoriatic fibroblasts associated with the regulation of gene expression is galectin-3. This protein is strongly expressed in epithelial cells, such as keratinocytes and skin fibroblasts, and is involved in the pathogenesis of inflammatory skin diseases by regulating the functions of immune cells, including lymphocytes [95,97]. Its decreased level in psoriatic keratinocytes has been shown to be a primary mechanism of cellular hyperproliferation, through the activation of the JNK pathway or the accumulation of neutrophils associated with S100A7-9 overexpression [98]. On the other hand, an increase in the levels of galectin-3 in the blood of psoriatic patients leads to inflammation and increased profibrotic activity [99]. However, the exact role of the downregulation of fibroblast galectin-3 in the development of psoriasis remains undefined.

Current evidence indicates that the development of psoriasis is likely associated with changes in the function of the immune system, as well as in skin cells. Psoriatic skin exfoliation is the result of changes in the metabolism of keratinocytes; however, metabolic changes in fibroblasts also contribute significantly to these symptoms.

## 4. Materials and Methods

### 4.1. Sample Collection and Preparation

Skin biopsy fragments were collected from five untreated patients with a diagnosis of psoriasis vulgaris. The patients were two men and three women; age range 27–48 years, mean 38. They were selected from a cohort of 70 patients because their skin lesions were the most characteristic of typical psoriasis. Biopsies were also taken from five healthy people who had moles, and the adjacent skin removed. These individuals forming a control group were sex-matched to the patient group. They had an age range of 28–50 years, mean 38.

The individuals selected for the patient group had had a diagnosis of plaque psoriasis for at least six months, with at least 10% of the total body surface area affected. The severity of psoriasis was assessed using the PASI score (Psoriasis Area and Severity Index) (median 18; range 12–25). None of the patients or healthy subjects had received topical, injectable or oral medications during the four weeks before the study. Individuals whose history indicated any other disorders were excluded from the study. None of the participants were smokers. All subjects gave their informed consent for inclusion before they participated in the study. The study was conducted in accordance with the Declaration of Helsinki, and the protocol was approved by the Local Bioethics Committee Medical University of Bialystok (Poland), No. R-I-002/502/2015 (17 December 2015). After the biopsy, skin fragments were taken for histopathological examination, and the remaining material was used for molecular analysis.

The samples were washed in PBS with 50 U/mL penicillin and 50 μg/mL streptomycin and incubated overnight at 4 °C in 1 mg/mL dispase to separate the epidermis from the dermis. The obtained dermis was sliced and placed in culture plates in fibroblast culture medium consisting of DMEM (Dulbecco’s Modified Eagle Medium), fetal bovine serum (10%) and penicillin (50 U/mL)/streptomycin (50 μg/mL). Samples were incubated in a humidified atmosphere of 5% CO_2_ at 37 °C until the fibroblasts emigrating from the slices reached full confluence.

Fibroblasts were collected from the plates by scraping on ice, and were suspending in buffer Tris-HCl (50 mM, pH 7.5, 4 °C) containing 0.1% SDS and protease inhibitor cocktail. All samples were lysed by sonification on ice. The total protein content in the cell lysates was measured using the Bradford assay [100].

### 4.2. SDS-PAGE and In-Gel Digestion

Each sample (containing 25 μg of protein) was mixed 1:1 with Laemmle buffer, containing 5% 2-mercaptoethanol, and heated at 100 °C for 7 min. After cooling to room temperature, the samples were separated on 12% Tris-Glycine SDS-PAGE gels. Gels were fixed for 1 h in methanol:acetic acid:water (4:1:5) and stained for 4 h with Coomassie Brilliant Blue R-250. All detected bands were cut out of the gel, sliced, and washed out of the dye by acetonitrile (ACN) and 25 mM AMBIC (ammonium bicarbonate). Proteins in each gel fragment were reduced for 1 h with 10 mM DTT, alkylated for 1 h with 50 mM iodoacetamide, and overnight in-gel digested with sequencing grade trypsin (Promega, Madison, WI, USA). The digestion was stopped by the addition of 10% FA (formic acid) and evaporated.

### 4.3. Liquid Chromatography-Mass Spectrometry (LC-MS/MS) Analysis

The dried peptides were dissolved in 5% ACN with 0.1% FA immediately before analysis and loaded onto a 150 mm x 75 µm PepMap RSLC capillary analytical C18 column with 2 μm particle size (LC Packings) using Ultimate 3000 HPLC system (Dionex, Idstein, Germany). Peptide separation was done at a flow rate of 0.300 µl/min, and the solvents gradient started at 3 min and was ramped to 60% Buffer B (90% ACN + 0.03% FA) over 60 min. Eluted peptides were analyzed using a Q Exactive HF mass spectrometer with a nanoelectrospray ionization source (ESI) (Thermo Fisher Scientific, Bremen, Germany). The mass spectrometer was externally calibrated and operated in positive and data-dependent modes. Survey MS scans were conducted in the 200–2000 m/z range, with a resolution of 120,000. The top ten most intense ions were fragmented with 30 eV collision energy on an HCD collision cell and analyzed with a resolution of 30,000. A 10 s dynamic exclusion window was applied, and an isolation window of 4 m/z was used to collect suitable tandem mass spectra. The obtained data were acquired with the Xcalibur software version 4.1 (Thermo Fisher Scientific, Bremen, Germany).

### 4.4. Protein Identification and Label-Free Quantification

For protein identification, Proteome Discoverer 2.0 (Thermo Fisher Scientific, Bremen, Germany) was used with the following search parameters: peptide mass tolerance set to 10 ppm, MS/MS mass tolerance set to 0.02 Da, up to two missed cleavages allowed, cysteine carbamidomethylation/carboxymethylation and methionine oxidation set as a dynamic modification, a minimum peptide length set to 6 amino acids, and the minimum number of identified unique peptides for each protein set to two peptides. Input data were searched against the UniProtKB-SwissProt database (taxonomy: Homo sapiens, release 2019-04). In the case of proteins that were identified in at least 60% of the examined samples from the control or psoriatic group, the missing values were estimated as half of the lowest recorded intensity (half minimum imputation). Other proteins were removed from the analysis as artefacts.

### 4.5. Statistical Analysis

The analysis of each sample was performed in three independent replicates. Data from individual protein label-free quantifications were log and Z-score transformed. Statistical analysis of data was performed using free available MetaboAnalyst 4.0 software (http://www.metaboanalyst.ca) (Xia Lab, Montreal, Quebec, Canada) [101], RStudio software (R version 3.6.2 (2019-12-12)) [102] and Perseus 1.6.10.43 [103]. Data were analyzed using the standard statistical analysis methods, including univariate analysis (one-way ANOVA), and only proteins with an FDR-corrected significant q-value were taken into account in the discussion. Protein molecular function and protein class were assigned according to the Gene Ontology database in the free available STRING version 11 (https://string-db.org/) (ELIXIR, Hinxton, Cambridgeshire, UK) [104].

## 5. Conclusions

As this study shows, fibroblast dysfunction results from the upregulation of pro-inflammatory factors and proteins with antioxidant properties, as well as factors involved in signal transduction and participating in proteolytic processes. The changes described may directly affect intercellular signaling and promote the hyperproliferation of epidermal cells. Therefore, a better understanding of their exact molecular mechanisms can contribute to the development of more effective pharmacotherapy.

## Supplementary Materials

The following are available online at https://www.mdpi.com/1422-0067/21/15/5363/s1, Table S1: Names and ID of proteins indicated in fibroblasts isolated from skin of psoriatic patients (*n* = 5) and healthy people (*n* = 5). Table S2: The p-values and fold change (FC) for individual statistically significant proteins indicated in fibroblasts isolated from skin of psoriatic patients (*n* = 5) and healthy people (*n* = 5).

## Abbreviations

4-**HNE**	4-hydroxynenenal
ACN	acetonitrile
AMBIC	ammonium bicarbonate
ANOVA	analysis of variance
C3	component 3
DMEM	Dulbecco’s Modified Eagle Medium
ERK	extracellular signal-regulated kinase
ESI	nanoelectrospray ionization
FA	formic acid
FDR	false discovery rate
HPLC	high performance liquid chromatography
IGF-I	insulin-like growth factor-I
IL-8	interleukin 8
JNK	c-Jun N-terminal kinase
KGF	keratinocyte growth factor
MAPK	mitogen-activated protein kinase
MDA	malondialdehyde
MS	mass spectrometry
NFκB	nuclear factor kappa-light-chain-enhancer of activated B cells
Nrf2	nuclear factor erythroid 2-related factor 2
PASI	Psoriasis Area and Severity Index
PCA	principal component analysis
PKC	protein kinase C
RANBP1	Ran-specific GTPase-activating protein 1
ROS	reactive oxygen species
SDS-PAGE	sodium dodecyl sulfate polyacrylamide gel electrophoresis
TGF-β1	transforming growth factor-β1
TNFα	tumor necrosis factor α.
