# Supplementary material for: Changes in Proteome of Fibroblasts Isolated from Psoriatic Skin Lesions
Source: Int J Mol Sci. 2020 Jul 28;21(15):5363. doi: 10.3390/ijms21155363 (PMC7432102; doi:10.3390/ijms21155363)
Supplement: Supplementary file 1 [file ijms-21-05363-s001.pdf]

Supplementary materials – list of contents

Supplementary table S1. Names and ID of proteins indicated in fibroblasts isolated from skin of psoriatic patients (n=5) and healthy people (n=5).

Supplementary table S2. The p-values and fold change (FC) for individual statistically significant proteins indicated in fibroblasts isolated from skin of psoriatic patients (n=5) and healthy people (n=5).

Table S1.

Names and ID of proteins indicated in fibroblasts isolated from skin of psoriatic patients (n=5) and healthy people (n=5). In the case of the proteins that have been identified in at least 60% of examined samples from psoriatic or control group, the missing values were estimated as the half of the lowest recorded intensity. Other proteins were removed from the analysis as artifacts. Abbreviations: n.d., not detected; +, detected;

ID	Protein name	Control	Psoriasis	Coverage [%]	No. of identified peptides	No. of identified unique peptides
A0A024QYX3	RNA binding motif (RNP1, RRM) protein 3	+	+	57.35	17	2
A0A024QZK8	Heterogeneous nuclear ribonucleoprotein H3 (2H9)	+	+	50.43	14	3
A0A024QZN9	Voltage-dependent anion channel 2	+	+	77.16	35	3
A0A024QZP7	Cyclin-dependent kinase 1	+	+	80.10	37	24
A0A024QZS4	Peptidyl-prolyl cis-trans isomerase	n.d.	+	79.70	36	8
A0A024QZY1	JTV1	+	+	51.61	15	4
A0A024R0E2	Cold shock domain containing E1	+	+	51.55	15	7
A0A024R0L6	Platelet-activating factor acetylhydrolase, isoform Ib	+	+	50.28	14	6
A0A024R0P9	Translocase of outer mitochondrial membrane 40 homolog	+	+	65.71	24	4
A0A024R0R4	SUMO-1 activating enzyme subunit 1	+	+	63.98	23	11
A0A024R0V4	Vasodilator-stimulated phosphoprotein isoform 1	+	+	76.35	34	5
A0A024R152	HCG28765	+	n.d.	49.02	14	13
A0A024R1A3	Ubiquitin-activating enzyme E1	+	+	70.63	28	8
A0A024R1K7	Tyrosine 3-monooxygenase/tryptophan 5-monooxygenase activation protein	+	+	68.93	26	11
A0A024R1N1	Myosin, heavy polypeptide 9, non-muscle	+	+	50.00	14	9
A0A024R1Q8	Ribosomal protein L23	+	+	88.06	42	8
A0A024R1S8	LIM and SH3 protein 1	+	+	87.73	40	12
A0A024R1V4	60S ribosomal protein L27	+	+	86.06	40	4
A0A024R203	Proteasome	+	+	85.93	39	4
A0A024R284	Ubiquilin 1	n.d.	+	85.36	39	7
A0A024R2M7	Oxidative-stress responsive 1	+	+	85.17	38	5
A0A024R2Q4	Ribosomal protein L15	+	+	51.11	15	4
A0A024R324	Ras homolog gene family, member A	+	+	50.93	15	3
A0A024R374	Cathepsin B	+	+	50.40	14	3
A0A024R394	Cysteine and histidine-rich domain	+	+	50.38	14	6
A0A024R3D8	Acetyltransferase component of pyruvate dehydrogenase complex	+	+	46.73	13	4
A0A024R3W7	Eukaryotic translation elongation factor 1 beta 2	+	+	46.73	13	5
A0A024R4E5	High density lipoprotein binding protein	+	+	50.00	14	8
A0A024R4U3	Tubulin tyrosine ligase-like family	+	+	68.75	26	6
A0A024R571	EH domain-containing protein 1	+	+	49.92	14	3
A0A024R5C5	Pyruvate carboxylase	+	+	49.79	14	6
A0A024R5Z7	Annexin isoform	+	+	49.72	14	2
A0A024R608	Ribosomal protein, large, P1	+	+	57.18	17	7
A0A024R6C9	Dihydrolipoamide S-succinyltransferase	+	+	49.44	14	5

A0A024R6W0	Enhancer of rudimentary homolog	+	+	49.32	14	12
A0A024R6W2	Aspartate aminotransferase isoform	+	+	72.87	30	7
A0A024R718	Nudix	+	+	72.73	30	4
A0A024R7B7	Pre-B-cell colony enhancing factor 1	+	+	72.56	30	14
A0A024R7T3	CDC37 cell division cycle 37	+	+	72.22	30	3
A0A024R7U6	Heterogeneous nuclear ribonucleoprotein F	+	+	48.90	14	10
A0A024R814	MCM4 minichromosome maintenance deficient 4	+	+	48.72	14	6
A0A024R8P8	Ribosomal protein L7	+	+	56.85	17	16
A0A024R8S5	Ribosomal protein L38	+	+	48.67	14	6
A0A024R8U5	Protein disulfide-isomerase	+	+	48.55	14	3
A0A024R8W0	Splicing factor, arginine/serine-rich 2	+	+	48.31	13	11
A0A024R904	DEAD (Asp-Glu-Ala-Asp)	+	+	48.15	13	7
A0A024R994	Calcyclin binding protein	+	+	46.09	13	4
A0A024R9W5	Copine III	+	+	46.06	13	2
A0A024RA52	Proteasome subunit alpha	+	+	70.85	28	4
A0A024RA75	3-hydroxyisobutyrate dehydrogenase	+	n.d.	70.80	28	10
A0A024RAD5	Dolichyl-diphosphooligosaccharide-protein glycosyltransferase	+	+	50.19	14	12
A0A024RAI1	ARP3 actin-related protein 3 homolog	+	+	49.07	14	13
A0A024RAM0	Transportin 1	+	+	61.98	21	2
A0A024RAN2	Calpastatin	+	+	48.29	13	4
A0A024RAZ7	Heterogeneous nuclear ribonucleoprotein A1	+	+	48.26	13	5
A0A024RB16	Family with sequence similarity 62	+	+	48.25	13	4
A0A024RBB7	Nucleosome assembly protein 1-like 1	+	+	44.53	12	2
A0A024RBE8	Solute carrier family 25 (Mitochondrial carrier phosphate carrier)	+	+	60.87	20	7
A0A024RBH2	Cytoskeleton-associated protein 4	+	+	44.12	12	2
A0A024RBS2	60S acidic ribosomal protein P0	+	+	44.47	12	6
A0A024RCN6	Valyl-tRNA synthetase	+	+	75.34	33	7
A0A024RD93	Phosphoribosylaminoimidazole carboxylase	+	+	45.19	12	3
A0A024RDE5	Ras-GTPase activating protein SH3 domain-binding protein 2	+	+	45.15	12	5
A0A024RDF6	Heterogeneous nuclear ribonucleoprotein D-like	+	+	45.09	12	9
A0A024RDR0	High-mobility group box 1	+	+	48.09	13	8
A0A024RDS1	Heat shock 105kDa/110kDa protein 1	+	+	47.91	13	4
A0A024RDT4	Lymphocyte cytosolic protein 1 (L-plastin)	+	+	47.78	13	3
A0A087WTP3	Far upstream element-binding protein 2	+	+	44.59	12	3
A0A087WUB9	Beta-catenin-like protein 1	+	+	44.44	12	8
A0A087WUT6	Eukaryotic translation initiation factor 5B	+	+	44.51	12	4
A0A087WUZ3	Spectrin beta chain, non-erythrocytic 1	+	+	61.66	21	3
A0A087X0X3	Heterogeneous nuclear ribonucleoprotein M	+	+	13.81	4	4
A0A087X1N8	Serpin B6	+	+	59.06	19	3
A0A087X1U6	Epiplakin	+	n.d.	45.79	13	3
A0A087X1Z3	Proteasome activator complex subunit 2	+	+	42.43	12	3
A0A087X2I1	26S protease regulatory subunit 10B	+	+	81.25	37	15
A0A0A0MRI2	Sorting nexin 6	+	n.d.	61.02	21	3
A0A0A0MSE2	Hydroxyacyl-coenzyme A dehydrogenase	+	+	42.76	12	5

A0A0A0MTN0	Cullin-2	+	+	42.64	12	5
A0A0A0MTS2	Glucose-6-phosphate isomerase	+	+	42.49	12	11
A0A0A6YYL6	Protein RPL17	+	+	42.35	12	9
A0A0B4J2C3	Translationally-controlled protein	+	+	65.63	24	24
A0A0C4DFU2	Superoxide dismutase	+	+	64.08	24	7
A0A0C4DG89	Probable ATP-dependent RNA helicase	+	+	42.93	12	2
A0A0C4DGQ5	Calpain small subunit 1	+	+	42.91	12	2
A0A0C4DH83	EH domain-containing protein 3	+	+	42.86	12	4
A0A0D9SF53	ATP-dependent RNA helicase	+	+	42.83	12	5
A0A0D9SFI9	Nuclear protein localization protein 4	+	+	13.83	4	4
A0A0G2JH68	Protein diaphanous homolog 1	+	+	41.01	11	2
A0A0G2JIW1	Heat shock 70 kDa protein 1B	+	+	41.00	11	3
A0A0G2JK23	Large proline-rich protein BAG6	+	+	40.99	11	5
A0A0K0K1K7	6-phosphogluconolactonase	+	+	42.14	12	2
A0A0S2Z2Z6	Annexin isoform	+	+	41.92	12	7
A0A0S2Z3L2	ATPase Ca+	+	+	41.88	12	5
A0A0S2Z3Y1	Lectin galactoside-binding soluble 3	+	+	41.61	12	11
A0A0S2Z410	Hydroxysteroid dehydrogenase 10	+	+	13.84	4	4
A0A0S2Z489	Proteasome 26S subunit	+	+	41.40	11	11
A0A0S2Z491	Nucleophosmin isoform 2	+	+	41.37	11	5
A0A0S2Z4A5	Minichromosome maintenance complex component 7	+	+	41.77	12	4
A0A0S2Z4G4	Tropomyosin 3 isoform 1	+	+	62.17	21	14
A0A0S2Z4J1	Hydroxysteroid (17-beta) dehydrogenase 4	+	+	42.40	12	3
A0A0S2Z4R1	Tyrosine-tRNA ligase	+	+	69.06	26	7
A0A0S2Z4Z9	Non-POU domain containing octamer-binding isoform 1	+	+	13.84	4	4
A0A0S2Z5J4	Adaptor-related protein complex 3	+	n.d.	35.06	9	5
A0A0S2Z5M8	ElaC homolog 2	+	+	19.46	5	3
A0A0U1RRM4	Polypyrimidine tract-binding protein 1	+	+	17.42	5	2
A0AVT1	Ubiquitin-like modifier-activating enzyme 6	+	+	73.23	31	2
A2A274	Aconitate hydratase, mitochondrial	+	n.d.	41.87	12	6
A2A3R6	40S ribosomal protein S6	+	+	71.69	29	9
A2RUM7	Ribosomal protein L5	+	+	56.73	17	9
A4D0V4	Capping protein (Actin filament)	+	+	41.57	12	3
A4D2P0	Ras-related C3 botulinum toxin substrate 1	+	+	41.52	12	3
A4QPB0	IQ motif containing GTPase activating protein 1	+	+	41.48	11	11
A5A3E0	POTE ankyrin domain family member F	+	+	17.45	5	5
A6NFX8	ADP-sugar pyrophosphatase	+	+	19.93	5	5
A8K0G3	AP complex subunit beta	+	+	19.47	5	5
A8K3S1	Glucosamine-6-phosphate isomerase	+	n.d.	14.12	4	4
A8K401	Prohibitin	+	+	17.55	5	5
A8K4D5	Kynureninase	+	+	18.11	5	5
A8K517	Ribosomal protein S23	+	+	57.02	17	10
A8K521	DNA helicase	+	+	19.56	5	5
A8K5K5	Eukaryotic translation initiation factor 3 subunit G	+	+	19.59	5	4

A8K5M4	Non-specific serine/threonine protein kinase	+	+	39.47	10	3
A8K7D9	Importin subunit alpha	+	+	17.55	5	5
A8K8D9	Glucose-6-phosphate 1-dehydrogenase	+	+	14.17	4	4
A8K8N7	Phosphoribosylformylglycinamidine synthase	+	+	38.44	10	4
A8K984	Structural maintenance of chromosomes protein	+	n.d.	63.44	23	7
A8MT02	Small nuclear ribonucleoprotein-associated proteins B	+	+	35.69	9	8
A8MUS3	60S ribosomal protein L23a	+	+	60.14	19	9
A8MXP9	Matrin-3	+	+	18.37	5	4
B0QY89	Eukaryotic translation initiation factor 3 subunit L	+	+	19.80	5	5
B0QZ18	Copine-1	+	+	19.93	5	5
B0ZBD0	40S ribosomal protein S19	+	+	40.70	11	3
B1AK88	Capping protein (Actin filament)	+	+	20.04	5	4
B2R4C0	60S ribosomal protein L18a	+	+	39.63	11	11
B2R4D5	Actin-related protein 2/3	+	+	36.55	9	3
B2R4R0	Histone H4	+	+	15.78	4	3
B2R4R9	HCG26477	+	+	14.46	4	3
B2R5W3	Poly [ADP-ribose] polymerase	+	n.d.	15.75	4	4
B2R657	Annexin isoform	+	+	20.17	5	3
B2R6F3	Splicing factor arginine/serine-rich 3	+	+	39.38	10	3
B2RAQ9	Proteasome subunit beta	+	+	17.63	5	4
B3KMS0	Condensin complex subunit 1	+	+	20.31	5	3
B3KS98	Eukaryotic translation initiation factor 3 subunit H	+	+	20.33	5	4
B3KSH1	Eukaryotic translation initiation factor 3 subunit F	+	+	20.40	5	5
B3KUY2	Prostaglandin E synthase 3 (Cytosolic)	+	+	17.61	5	3
B4DHQ3	Phosphoserine aminotransferase	+	+	38.49	10	8
B4DR52	Histone H2B	+	+	15.69	4	4
B5BU01	Eukaryotic translation initiation factor 2	+	n.d.	20.41	5	5
B5BU83	Stathmin	+	+	63.37	23	20
B5BUB1	RuvB-like helicase	+	+	34.09	8	4
B5BUB5	Autoantigen La	+	+	20.47	5	5
B5BUE6	ATP-dependent RNA helicase DDX5	+	+	20.48	5	5
B5ME19	Eukaryotic translation initiation factor 3 subunit C	+	+	20.52	5	5
B7Z3K9	Fructose-bisphosphate aldolase	+	+	20.56	5	5
B7Z4C8	60S ribosomal protein L31	+	+	37.42	10	8
B7Z6L0	Annexin isoform	+	+	20.60	5	5
B9EKV4	Aldehyde dehydrogenase 9 family	+	+	20.79	5	5
B9VP24	60 kDa chaperonin	+	+	82.26	38	5
D3DPU2	Adenylyl cyclase-associated protein	+	+	20.82	5	5
D3DRX6	Kinesin-like protein	+	+	19.17	5	5
D3DUW5	Dynamin 1-like	+	+	20.80	5	5
D6RER5	Septin-11	+	+	34.43	9	4
E5KNY5	Leucine-rich PPR-motif	+	+	18.35	5	4
E5RHG6	Tubulin-specific chaperone A	+	+	68.79	26	10
E7EPK1	Septin-7	+	+	34.44	9	7

E7EVA0	Microtubule-associated protein	+	+	18.54	5	5
E9PB61	THO complex subunit 4	+	+	37.77	10	2
E9PCR7	2-oxoglutarate dehydrogenase, mitochondrial	+	+	40.31	11	4
E9PFW3	AP-2 complex	+	+	20.94	5	5
E9PRY8	Elongation factor 1-delta	+	+	20.96	5	4
F4ZW62	NF45	+	+	39.47	10	2
F5H2F4	C-1-tetrahydrofolate synthase, cytoplasmic	+	+	21.00	5	4
F5H5D3	Tubulin alpha-1C	+	+	45.60	12	2
F6WQW2	Ran-specific GTPase-activating protein	+	+	32.75	8	5
F8VV71	Ubiquitin-conjugating enzyme E2 N	+	+	72.88	31	3
F8W727	60S ribosomal protein L32	+	+	37.36	10	8
F8WCF6	Actin-related protein 2/3 complex subunit 4	+	+	35.19	9	4
G3V180	Dipeptidyl peptidase 3	+	+	20.98	5	5
G3V4C1	Heterogeneous nuclear ribonucleoproteins C1/C2	+	+	15.64	4	3
G8JLA2	Myosin light polypeptide 6	+	+	18.95	5	4
G8JLB6	Heterogeneous nuclear ribonucleoprotein H	+	+	14.49	4	3
H0Y8C6	Importin-5	+	+	17.61	5	3
H6VRG1	Keratin 1	+	+	17.92	5	4
H7C2I1	Protein arginine N-methyltransferase 1	+	+	18.11	5	5
H7C2Q3	26S proteasome non-ATPase regulatory subunit 2	+	+	67.91	25	5
H9ZYJ2	Thioredoxin	+	+	37.63	10	10
I3L0A0	HCG2044781	+	+	14.44	4	3
J3K000	PEPD protein	+	+	21.19	5	5
J3KN16	Proteasome-associated protein	+	+	18.08	5	5
J3KN67	Tropomyosin alpha-3	+	+	62.24	22	3
J3KPF3	4F2 cell-surface antigen heavy chain	+	+	84.35	38	5
J3KQ32	Obg-like ATPase 1	+	+	21.17	5	5
J3KQE5	GTP-binding nuclear protein Ran	+	+	14.43	4	4
J3KRF5	Clathrin heavy chain 1	+	+	21.12	5	5
J3KTL2	Serine/arginine-rich-splicing factor 1	+	+	34.55	9	3
J3QQ67	60S ribosomal protein L18	+	+	39.76	11	6
J9R021	Eukaryotic translation initiation factor 3	+	+	17.11	5	4
K7EKE6	Lon protease homolog	+	+	18.39	5	5
K7ELC2	40S ribosomal protein S15	+	+	80.79	37	3
K7ER00	Phenylalanine-tRNA ligase alpha	+	+	21.33	6	3
L0R5C4	Alternative protein POTEM	+	n.d.	17.25	5	5
L0R849	Alternative protein EDARADD	+	+	17.33	5	5
L7RSM2	Mitogen-activated protein kinase	+	+	18.87	5	4
M0QYS1	60S ribosomal protein L13a	+	+	39.77	11	7
M0R2B7	DNA polymerase	+	n.d.	17.38	5	2
O00154	Cytosolic acyl coenzyme A thioester hydrolase	+	+	17.38	5	5
O00231	26S proteasome non-ATPase regulatory subunit 11	+	+	89.01	43	5
O00469	Procollagen-lysine,2-oxoglutarate 5-dioxygenase 2	+	+	17.52	5	4
O00487	26S proteasome non-ATPase regulatory subunit 14	+	+	40.91	11	10

O14980	Exportin-1	+	+	18.37	5	4
O15143	Actin-related protein 2/3 complex subunit 1B	+	+	36.42	9	8
O15144	Actin-related protein 2/3 complex subunit 2	+	+	36.41	9	4
O15371	Eukaryotic translation initiation factor 3 subunit D	+	+	18.34	5	5
O15397	Importin-8	+	n.d.	17.62	5	5
O43143	Pre-mRNA-splicing factor ATP-dependent RNA helicase DHX15	+	+	17.49	5	4
O43242	26S proteasome non-ATPase regulatory subunit 3	+	+	40.69	11	2
O43390	Heterogeneous nuclear ribonucleoprotein R	+	+	14.79	4	3
O43615	Mitochondrial import inner membrane translocase subunit TIM44	+	n.d.	18.85	5	4
O43707	Alpha-actinin-4	+	+	19.74	5	4
O43776	Asparagine-tRNA ligase, cytoplasmic	+	+	19.77	5	4
O60506	Heterogeneous nuclear ribonucleoprotein Q	+	+	14.75	4	3
O60664	Perilipin-3	+	+	21.32	5	5
O60701	UDP-glucose 6-dehydrogenase	+	+	73.53	31	5
O60888	Protein CutA	+	+	17.92	5	4
O75083	WD repeat-containing protein 1	+	+	78.17	35	4
O75367	Core histone macro-H2A	+	+	21.19	5	5
O75369	Filamin-B	+	+	21.20	5	4
O75390	Citrate synthase, mitochondrial	+	+	18.85	5	4
O75396	Vesicle-trafficking protein SEC22b	+	n.d.	76.72	35	5
O75533	Splicing factor 3B subunit 1	+	+	38.95	10	8
O75643	U5 small nuclear ribonucleoprotein 200 kDa helicase	+	+	69.58	27	8
O75937	DnaJ homolog subfamily C member 8	+	+	21.32	5	5
O76003	Glutaredoxin-3	n.d.	+	21.33	6	3
O76021	Ribosomal L1 domain-containing protein 1	+	+	32.93	8	5
O95373	Importin-7	+	+	18.08	5	5
O95433	Activator of 90 kDa heat shock protein ATPase homolog 1	+	+	35.11	9	4
O95678	Keratin, type II cytoskeletal 75	+	n.d.	21.66	6	4
O96019	Actin-like protein 6A	+	n.d.	21.71	6	5
P00338	L-lactate dehydrogenase A	+	+	21.72	6	4
P00387	NADH-cytochrome b5 reductase 3	+	n.d.	21.74	6	4
P00492	Hypoxanthine-guanine phosphoribosyltransferase	+	+	21.76	6	5
P00558	Phosphoglycerate kinase 1	+	+	21.84	6	5
P01375	Tumor necrosis factor alfa	n.d.	+	63.12	23	7
P04075	Fructose-bisphosphate aldolase A	+	+	22.37	6	3
P04083	Annexin A1	+	+	22.39	6	4
P04181	Ornithine aminotransferase, mitochondrial	+	+	22.47	6	3
P04259	Keratin, type II cytoskeletal 6B	+	+	22.63	6	5
P04350	Tubulin beta-4A	+	+	68.42	26	12
P04406	Glyceraldehyde-3-phosphate dehydrogenase	+	+	21.17	5	5
P04843	Dolichyl-diphosphooligosaccharide-protein glycosyltransferase subunit 1	+	+	21.56	6	4
P04844	Dolichyl-diphosphooligosaccharide-protein glycosyltransferase subunit 2	+	+	21.64	6	5
P05023	Sodium/potassium-transporting ATPase subunit alpha-1	+	+	60.27	20	3
P05109	Protein S100-A8	+	+	18.54	5	5

P05141	ADP/ATP translocase 2	+	+	21.94	6	4
P05198	Eukaryotic translation initiation factor 2 subunit 1	+	+	21.50	6	2
P05556	Integrin beta-1	+	+	17.69	5	3
P05787	Keratin, type II cytoskeletal 8	+	+	18.34	5	5
P06702	Protein S100-A9	+	+	52.04	15	8
P06733	Alpha-enolase	+	+	21.95	6	3
P06737	Glycogen phosphorylase	+	+	14.31	4	4
P06899	Histone H2B type 1	+	+	15.75	4	4
P07205	Phosphoglycerate kinase 2	+	+	22.22	6	6
P07384	Calpain-1 catalytic subunit	+	+	21.97	6	5
P07737	Profilin-1	+	+	17.55	5	5
P07741	Adenine phosphoribosyltransferase	+	+	37.60	10	3
P07814	Bifunctional glutamate/proline-tRNA ligase	n.d.	+	22.03	6	4
P07900	Heat shock protein HSP 90-alpha	+	+	15.03	4	4
P07954	Fumarate hydratase, mitochondrial	+	+	22.12	6	4
P08134	Rho-related GTP-binding protein RhoC	+	+	22.63	6	5
P08238	Heat shock protein HSP 90-beta	+	+	22.64	6	4
P08263	Glutathione S-transferase A1	n.d.	+	22.67	6	4
P08708	40S ribosomal protein S17	+	+	22.67	6	4
P08727	Keratin, type I cytoskeletal 19	+	+	22.85	6	5
P08729	Keratin, type II cytoskeletal 7	+	+	22.90	6	4
P09382	Galectin-1	+	+	22.94	6	4
P09467	Fructose-1,6-bisphosphatase	+	+	22.16	6	5
P09622	Dihydrolipoyl dehydrogenase, mitochondrial	+	+	22.22	6	6
P09661	U2 small nuclear ribonucleoprotein A	+	+	69.49	26	3
P09960	Leukotriene A-4 hydrolase	+	+	22.99	6	6
P0CG39	POTE ankyrin domain family member J	+	n.d.	23.04	6	4
P10644	cAMP-dependent protein kinase type I-alpha regulatory subunit	+	+	23.05	6	5
P10768	S-formylglutathione hydrolase	+	+	23.08	6	4
P10809	60 kDa heat shock protein, mitochondrial	+	+	39.91	11	11
P11177	Pyruvate dehydrogenase E1 component subunit beta	+	+	32.54	8	4
P11216	Glycogen phosphorylase	+	+	14.36	4	4
P11279	Lysosome-associated membrane glycoprotein 1	+	+	18.36	5	4
P11908	Ribose-phosphate pyrophosphokinase 2	+	+	24.81	6	4
P11940	Polyadenylate-binding protein 1	+	+	24.83	6	6
P12004	Proliferating cell nuclear antigen	+	+	24.86	6	4
P12081	Histidine-tRNA ligase, cytoplasmic	+	+	25.00	6	6
P12268	Inosine-5'-monophosphate dehydrogenase 2	+	+	17.63	5	4
P12429	Annexin A3	+	+	17.21	5	3
P12814	Alpha-actinin-1	+	+	17.21	5	5
P13489	Ribonuclease inhibitor	+	+	33.65	8	8
P13639	Elongation factor 2	+	+	22.22	6	4
P13645	Keratin, type I cytoskeletal 10	+	+	17.99	5	2
P13667	Protein disulfide-isomerase A4	+	+	17.99	5	2

P13797	Plastin-3	+	+	38.80	10	7
P13804	Electron transfer flavoprotein subunit alpha	+	+	22.22	6	4
P14174	Macrophage migration inhibitory factor	+	+	23.49	6	6
P14625	Endoplasmin	+	+	23.51	6	5
P14866	Heterogeneous nuclear ribonucleoprotein L	+	+	23.57	6	4
P15121	Aldose reductase	+	+	23.64	6	4
P15880	40S ribosomal protein S2	+	+	71.32	29	3
P16070	CD44 antigen	+	+	23.19	6	4
P16152	Carbonyl reductase [NADPH] 1	+	+	22.22	6	4
P16403	Histone H1.2	+	+	15.43	4	4
P16930	Fumarylacetoacetase	+	+	17.23	5	5
P17174	Aspartate aminotransferase, cytoplasmic	+	+	17.24	5	5
P17812	CTP synthase 1	+	+	21.95	6	3
P17931	Galectin-3	+	n.d.	25.14	6	5
P17980	26S protease regulatory subunit 6A	+	+	25.19	6	5
P17987	T-complex protein 1 subunit alpha	+	+	25.22	6	5
P18077	60S ribosomal protein L35a	+	n.d.	25.37	7	3
P18085	ADP-ribosylation factor 4	+	n.d.	25.41	7	3
P19012	Keratin, type I cytoskeletal 15	+	n.d.	25.41	7	3
P19338	Nucleolin	+	+	25.41	7	2
P20290	Transcription factor BTF3	+	+	59.67	19	12
P20618	Proteasome subunit beta type-1	+	+	17.69	5	3
P20700	Lamin-B1	+	+	18.28	5	4
P21333	Filamin-A	+	+	23.26	6	4
P21399	Cytoplasmic aconitate hydratase	+	+	19.56	5	5
P21796	Voltage-dependent anion-selective channel protein 1	+	+	77.27	35	4
P22626	Heterogeneous nuclear ribonucleoproteins A2/B1	+	+	15.61	4	4
P23246	Splicing factor, proline- and glutamine-rich	+	+	36.36	9	2
P23284	Peptidyl-prolyl cis-trans isomerase B	+	+	21.20	5	4
P23381	Tryptophan-tRNA ligase, cytoplasmic	+	+	55.10	16	5
P23396	40S ribosomal protein S3	+	+	40.97	11	5
P23526	Adenosylhomocysteinase	+	+	37.66	10	6
P23528	Cofilin-1	+	+	19.12	5	3
P23921	Ribonucleoside-diphosphate reductase large subunit	+	+	33.73	8	5
P24752	Acetyl-CoA acetyltransferase, mitochondrial	+	+	36.90	9	3
P25398	40S ribosomal protein S12	+	+	40.85	11	10
P25786	Proteasome subunit alpha type-1	+	+	17.62	5	5
P25788	Proteasome subunit alpha type-3	+	+	19.31	5	5
P25789	Proteasome subunit alpha type-4	+	+	25.71	7	2
P26006	Integrin alpha-3	+	+	25.77	7	6
P26368	Splicing factor U2AF 65 kDa	+	+	25.87	7	2
P26373	60S ribosomal protein L13	+	+	26.03	7	3
P26583	High mobility group protein B2	n.d.	+	26.10	7	6
P26639	Threonine-tRNA ligase, cytoplasmic	+	+	26.15	7	5

P26641	Elongation factor 1-gamma	+	+	13.79	4	4
P27348	14-3-3 protein theta	+	+	82.02	37	5
P27635	60S ribosomal protein L10	+	+	39.79	11	6
P27694	Replication protein A 70 kDa DNA-binding subunit	+	+	33.26	8	3
P27695	DNA-(apurinic or apyrimidinic site) lyase	+	+	14.00	4	3
P27708	CAD protein	+	+	14.12	4	4
P27824	Calnexin	+	+	26.42	7	5
P28070	Proteasome subunit beta type-4	+	+	26.45	7	6
P28072	Proteasome subunit beta type-6	+	+	26.59	7	6
P28074	Proteasome subunit beta type-5	+	+	27.52	7	2
P28838	Cytosol aminopeptidase	+	+	27.72	7	3
P29401	Transketolase	+	+	27.92	7	4
P30040	Endoplasmic reticulum resident protein 29	+	+	28.04	7	6
P30048	Thioredoxin-dependent peroxide reductase, mitochondrial	+	+	28.07	7	3
P30050	60S ribosomal protein L12	+	+	79.11	36	6
P30085	UMP-CMP kinase	+	+	74.14	31	23
P30101	Protein disulfide-isomerase A3	+	+	14.00	4	4
P30153	Serine/threonine-protein phosphatase 2A 65 kDa	+	+	34.61	9	3
P30419	Glycylpeptide N-tetradecanoyltransferase 1	+	+	27.01	7	4
P30837	Aldehyde dehydrogenase, mitochondrial	+	+	27.09	7	2
P31153	S-adenosylmethionine synthase isoform type-2	+	+	27.31	7	5
P31689	DnaJ homolog subfamily A member 1	+	+	28.45	7	4
P31939	Bifunctional purine biosynthesis protein PURH	+	+	28.57	7	4
P31947	14-3-3 protein sigma	+	+	28.57	7	4
P32119	Peroxiredoxin-5, mitochondrial	+	+	28.60	7	4
P34897	Serine hydroxymethyltransferase, mitochondrial	+	+	28.80	7	2
P35222	Catenin beta-1	+	n.d.	28.84	7	6
P35268	60S ribosomal protein L22	+	+	29.05	7	6
P35527	Keratin, type I cytoskeletal 9	n.d.	+	29.12	7	3
P35580	Myosin-10	+	+	29.14	7	3
P35609	Alpha-actinin-2	+	+	30.16	8	3
P35659	Protein DEK	+	+	30.33	8	2
P35908	Keratin, type II cytoskeletal 2	n.d.	+	30.41	8	2
P35998	26S protease regulatory subunit 7	+	+	94.28	44	4
P36507	Dual specificity mitogen-activated protein kinase kinase 2	n.d.	+	13.99	4	2
P36542	ATP synthase subunit gamma, mitochondrial	+	+	31.71	8	5
P36551	Oxygen-dependent coproporphyrinogen-III oxidase	+	+	31.74	8	5
P36871	Phosphoglucomutase-1	+	+	31.84	8	3
P37802	Transgelin-2	+	+	32.02	8	7
P37837	Transaldolase	+	+	59.59	19	10
P38117	Electron transfer flavoprotein subunit beta	n.d.	+	14.20	4	4
P38606	V-type proton ATPase catalytic subunit A	+	+	77.38	35	3
P38646	Stress-70 protein, mitochondrial	+	+	63.39	23	3
P39023	60S ribosomal protein L3	+	+	37.42	10	8

P40227	T-complex protein 1 subunit zeta	+	+	58.87	18	4
P40763	Signal transducer and activator of transcription 3	+	+	35.02	9	2
P40925	Malate dehydrogenase, cytoplasmic	+	+	13.62	4	4
P40926	Malate dehydrogenase, mitochondrial	+	+	13.92	4	4
P41091	Eukaryotic translation initiation factor 2 subunit 3	+	+	13.98	4	4
P41250	Glycine-tRNA ligase	+	+	33.26	8	4
P42766	60S ribosomal protein L35	+	+	33.33	8	6
P43246	DNA mismatch repair protein Msh2	+	+	33.45	8	3
P43686	26S protease regulatory subunit 6B	+	+	33.47	8	2
P45974	Ubiquitin carboxyl-terminal hydrolase 5	+	+	70.00	27	2
P46060	Ran GTPase-activating protein 1	+	+	32.73	8	5
P46776	60S ribosomal protein L27a	+	+	37.95	10	10
P46781	40S ribosomal protein S9	+	+	84.65	38	5
P46783	40S ribosomal protein S10	+	+	40.85	11	11
P46934	E3 ubiquitin-protein ligase NEDD4	n.d.	+	14.54	4	4
P47897	Glutamine-tRNA ligase	+	+	14.63	4	4
P48960	CD97 antigen	+	+	14.69	4	4
P49321	Nuclear autoantigenic sperm protein	+	+	39.60	10	6
P49327	Fatty acid synthase	+	+	34.09	8	8
P49411	Elongation factor Tu, mitochondrial	+	+	34.17	8	8
P49588	Alanine-tRNA ligase, cytoplasmic	+	+	34.21	9	4
P49720	Proteasome subunit beta type-3	+	+	34.22	9	6
P49721	Proteasome subunit beta type-2	+	+	34.24	9	4
P49736	DNA replication licensing factor MCM2	+	+	34.29	9	2
P49748	Very long-chain specific acyl-CoA dehydrogenase, mitochondrial	+	+	76.51	34	3
P49756	RNA-binding protein 25	n.d.	+	33.52	8	2
P49773	Histidine triad nucleotide-binding protein 1	+	+	14.81	4	4
P49915	GMP synthase [glutamine-hydrolyzing]	+	+	14.86	4	3
P50454	Serpin H1	+	+	59.22	19	8
P50502	Hsc70-interacting protein	+	+	14.88	4	4
P50570	Dynamin-2	+	+	14.99	4	4
P50990	T-complex protein 1 subunit theta	+	+	58.79	18	3
P50991	T-complex protein 1 subunit delta	+	+	58.45	18	7
P51148	Ras-related protein Rab-5C	n.d.	+	31.60	8	5
P51149	Ras-related protein Rab-7a	+	+	33.22	8	4
P51991	Heterogeneous nuclear ribonucleoprotein A3	+	+	14.99	4	3
P52209	6-phosphogluconate dehydrogenase, decarboxylating	+	+	37.29	10	10
P52566	Rho GDP-dissociation inhibitor 2	n.d.	+	32.81	8	5
P52895	Aldo-keto reductase family 1 member C2	+	+	35.19	9	7
P53618	Coatomer subunit beta	+	+	35.22	9	5
P53621	Coatomer subunit alpha	+	+	35.24	9	7
P53992	Sec24C	+	+	35.56	9	5
P54136	Arginine-tRNA ligase, cytoplasmic	+	+	35.66	9	4
P54578	Ubiquitin carboxyl-terminal hydrolase 14	+	+	69.66	27	6

P54709	Sodium/potassium-transporting ATPase subunit beta-3	+	+	60.70	20	3
P54727	UV excision repair protein RAD23 homolog B	+	+	74.31	32	5
P54819	Adenylate kinase 2, mitochondrial	+	+	37.63	10	8
P54886	Delta-1-pyrroline-5-carboxylate synthase	+	+	20.52	5	5
P55010	Eukaryotic translation initiation factor 5	+	+	15.04	4	4
P55060	Exportin-2	+	+	20.47	5	5
P55084	Trifunctional enzyme subunit beta	n.d.	+	62.00	21	14
P55263	Adenosine kinase	+	+	37.50	10	6
P55786	Puromycin-sensitive aminopeptidase	+	+	32.53	8	3
P55884	Eukaryotic translation initiation factor 3 subunit B	+	+	16.00	4	4
P56537	Eukaryotic translation initiation factor 6	+	+	19.77	5	4
P60174	Triosephosphate isomerase	+	+	54.51	16	6
P60228	Eukaryotic translation initiation factor 3 subunit E	+	+	19.74	5	4
P60842	Eukaryotic initiation factor 4A-I	+	+	20.56	5	5
P60866	40S ribosomal protein S20	+	+	71.05	28	17
P61026	Ras-related protein Rab-10	+	+	31.53	8	7
P61081	NEDD8-conjugating enzyme Ubc12	+	+	15.59	4	4
P61160	Actin-related protein 2	+	+	36.63	9	3
P61163	Alpha-centractin	+	+	36.25	9	5
P61204	ADP-ribosylation factor 3	+	+	36.36	9	4
P61221	ATP-binding cassette sub-family E	+	+	36.36	9	4
P61247	40S ribosomal protein S3a	+	+	63.12	23	6
P61254	60S ribosomal protein L26	+	+	37.81	10	8
P61604	10 kDa heat shock protein, mitochondrial	+	+	60.27	20	3
P61970	Nuclear transport factor 2	+	+	36.97	10	3
P61981	14-3-3 protein gamma	+	+	37.05	10	4
P62081	40S ribosomal protein S7	+	+	37.18	10	6
P62136	Serine/threonine-protein phosphatase PP1-alpha catalytic subunit	+	+	37.25	10	3
P62195	26S protease regulatory subunit 8	+	+	91.61	44	3
P62244	40S ribosomal protein S15a	+	+	80.79	37	37
P62249	40S ribosomal protein S16	+	+	40.37	11	5
P62258	14-3-3 protein epsilon	+	+	60.16	20	9
P62263	40S ribosomal protein S14	+	+	66.53	24	17
P62269	40S ribosomal protein S18	+	+	40.74	11	7
P62277	40S ribosomal protein S13	+	+	40.74	11	3
P62314	Small nuclear ribonucleoprotein Sm D1	+	+	36.03	9	2
P62318	Small nuclear ribonucleoprotein Sm D3	+	+	36.06	9	5
P62424	60S ribosomal protein L7a	+	+	36.10	9	6
P62495	Eukaryotic peptide chain release factor subunit 1	+	+	36.15	9	4
P62701	40S ribosomal protein S4	+	+	72.19	30	25
P62847	40S ribosomal protein S24	+	+	64.44	24	3
P62851	40S ribosomal protein S25	+	+	35.89	9	9
P62854	40S ribosomal protein S26	+	+	35.92	9	5
P62888	60S ribosomal protein L30	+	+	36.01	9	4

P62906	60S ribosomal protein L10a	+	+	36.01	9	3
P62913	60S ribosomal protein L11	+	+	79.40	36	5
P62917	60S ribosomal protein L8	+	+	37.91	10	4
P62942	Peptidyl-prolyl cis-trans isomerase FKBP1A	+	+	22.03	6	4
P62979	Ubiquitin-40S ribosomal protein S27a	+	+	70.43	27	3
P63104	14-3-3 protein zeta/delta	+	+	81.46	37	4
P67775	Serine/threonine-protein phosphatase 2A catalytic subunit alpha	+	+	34.68	9	3
P67809	Nuclease-sensitive element-binding protein 1	+	+	22.16	6	5
P67936	Tropomyosin alpha-4 chain	+	+	54.82	16	9
P68032	Actin, alpha cardiac muscle 1	+	+	36.76	9	4
P68363	Tubulin alpha-1B chain	+	+	45.44	12	4
P68366	Tubulin alpha-4A chain	+	+	45.78	13	6
P68371	Tubulin beta-4B chain	+	+	68.57	26	4
P78371	T-complex protein 1	+	+	62.77	22	15
P78527	DNA-dependent protein kinase catalytic subunit	+	+	15.78	4	3
P83731	60S ribosomal protein L24	+	+	39.91	11	10
P84098	60S ribosomal protein L19	+	+	40.00	11	5
P99999	Cytochrome c	+	+	40.07	11	5
Q00653	Nuclear factor NF-kappa-B p100 subunit	n.d.	+	40.15	11	10
Q00839	Heterogeneous nuclear ribonucleoprotein U	+	+	40.20	11	4
Q01813	ATP-dependent 6-phosphofructokinase, platelet type	+	+	28.71	7	3
Q02790	Peptidyl-prolyl cis-trans isomerase FKBP4	+	+	16.01	4	4
Q04837	Single-stranded DNA-binding protein, mitochondrial	+	+	35.05	9	6
Q05519	Serine/arginine-rich splicing factor 11	+	+	38.18	10	7
Q05639	Elongation factor 1-alpha 2	+	+	38.19	10	6
Q05655	Protein kinase C delta	+	n.d.	38.21	10	4
Q05D08	PA2G4 protein	+	+	38.39	10	6
Q06830	Peroxiredoxin-1	+	+	22.25	6	4
Q07021	Complement component 1 Q, mitochondrial	+	+	40.40	11	8
Q07666	KH domain-containing, RNA-binding, signal transduction-associated protein 1	+	+	40.41	11	11
Q07960	Rho GTPase-activating protein 1	n.d.	+	40.43	11	5
Q08211	ATP-dependent RNA helicase A	+	+	40.53	11	9
Q08AJ9	Histone H2A	n.d.	+	40.53	11	9
Q08J23	tRNA (cytosine(34)-C(5))-methyltransferase	+	+	54.67	16	7
Q09161	Nuclear cap-binding protein subunit 1	+	+	23.78	6	5
Q09666	Neuroblast differentiation-associated protein AHNAK	+	+	23.85	6	3
Q12904	Aminoacyl tRNA synthase complex-interacting multifunctional protein 1 OS=Homo sapiens GN=AIMP1 PE=1 SV=2	+	+	24.13	6	6
Q12906	Interleukin enhancer-binding factor 3	+	+	13.67	4	3
Q12931	Heat shock protein 75 kDa, mitochondrial	+	+	22.26	6	4
Q13011	Delta(3,5)-Delta(2,4)-dienoyl-CoA isomerase, mitochondrial	n.d.	+	22.29	6	6
Q13057	Bifunctional coenzyme A synthase	+	+	22.35	6	6
Q13148	TAR DNA-binding protein 43	+	+	62.63	22	5
Q13151	Heterogeneous nuclear ribonucleoprotein A0	+	+	23.71	6	5

Q13185	Chromobox protein homolog 3	+	+	23.72	6	3
Q13200	26S proteasome non-ATPase regulatory subunit 2	+	+	67.91	25	8
Q13283	Ras GTPase-activating protein-binding protein 1	+	+	32.77	8	4
Q13435	Splicing factor 3B subunit 2	+	+	39.22	10	8
Q13501	Sequestosome-1	+	+	34.45	9	4
Q13620	Cullin-4B	+	+	23.68	6	3
Q13813	Spectrin alpha chain, non-erythrocytic 1	+	+	61.54	21	20
Q13838	Spliceosome RNA helicase DDX39B	+	+	61.74	21	4
Q14019	Coactosin-like protein	+	+	24.37	6	6
Q14103	Heterogeneous nuclear ribonucleoprotein D0	+	+	24.46	6	4
Q14204	Cytoplasmic dynein 1 heavy chain 1	+	+	26.34	7	4
Q14222	EEF1A protein	+	+	26.35	7	5
Q14240	Eukaryotic initiation factor 4A-II	+	+	26.41	7	3
Q14247	Src substrate cortactin	+	+	63.29	23	7
Q14315	Filamin-C	+	+	24.17	6	4
Q14376	UDP-glucose 4-epimerase	+	+	73.48	31	23
Q14444	Caprin-1	n.d.	+	24.19	6	4
Q14498	RNA-binding protein 39	+	+	57.41	17	5
Q14566	DNA replication licensing factor MCM6	+	+	24.57	6	4
Q14683	Structural maintenance of chromosomes protein 1A	n.d.	+	35.88	9	3
Q14764	Major vault protein	+	+	25.45	7	6
Q14974	Importin subunit beta-1	+	+	25.46	7	4
Q15008	26S proteasome non-ATPase regulatory subunit 6	+	+	13.79	4	3
Q15019	Septin-2	+	+	42.96	12	5
Q15046	Lysine-tRNA ligase	+	+	43.01	12	3
Q15056	Eukaryotic translation initiation factor 4H	+	+	43.15	12	2
Q15084	Protein disulfide-isomerase A6	+	+	43.34	12	3
Q15149	Plectin	+	+	43.36	12	9
Q15181	Inorganic pyrophosphatase	+	+	43.42	12	8
Q15293	Reticulocalbin-1	+	+	43.72	12	6
Q15370	Transcription elongation factor B polypeptide 2	+	+	59.62	19	12
Q15393	Splicing factor 3B subunit 3	+	+	39.33	10	5
Q15417	Calponin-3	+	+	24.74	6	3
Q15436	Protein transport protein Sec23A	+	+	52.57	15	8
Q15437	Protein transport protein Sec23B	+	+	52.59	15	12
Q15459	Splicing factor 3A subunit 1	+	+	38.86	10	3
Q15637	Splicing factor 1	+	+	35.88	9	2
Q15642	Cdc42-interacting protein 4	+	+	25.05	6	2
Q15691	Microtubule-associated protein RP/EB family member 1	+	+	25.10	6	5
Q15717	ELAV-like protein 1	+	+	25.13	6	2
Q15758	Neutral amino acid transporter B(0)	+	+	39.44	10	3
Q15785	Mitochondrial import receptor subunit TOM34	+	+	24.75	6	4
Q15942	Zyxin	+	+	78.57	35	10
Q16222	UDP-N-acetylhexosamine pyrophosphorylase	+	+	73.99	31	8

Q16236	Nuclear factor erythroid 2-related factor 2	+	+	39.72	11	7
Q16531	DNA damage-binding protein 1	+	+	25.64	7	2
Q16576	Histone-binding protein RBBP7	+	+	25.65	7	7
Q16629	Serine/arginine-rich splicing factor 7	+	+	34.51	9	4
Q16881	Thioredoxin reductase 1, cytoplasmic	+	+	71.65	29	15
Q1KMD3	Heterogeneous nuclear ribonucleoprotein U-like protein 2	+	+	24.59	6	5
Q2NKY5	TUBB6 protein	+	+	45.22	12	2
Q32P28	Prolyl 3-hydroxylase 1	+	+	25.50	7	2
Q32Q12	Nucleoside diphosphate kinase	+	+	25.54	7	3
Q3ZCR3	TUBB3 protein	+	+	55.34	16	3
Q4LE36	ACLY variant protein	+	+	36.95	10	3
Q4LE58	EIF4G1 variant protein	+	+	13.92	4	4
Q4W4Y1	Dopamine receptor interacting protein 4	+	+	25.89	7	3
Q53F64	Heterogeneous nuclear ribonucleoprotein AB	+	+	26.74	7	6
Q53FN7	BZW1 protein variant	+	+	26.84	7	9
Q53G35	Phosphoglycerate mutase 1	+	+	26.92	7	3
Q53GR7	Solute carrier family 25, member 13	+	n.d.	61.02	21	12
Q53H88	Dynactin 2 variant	+	+	26.63	7	6
Q53HB3	Proteasome 26S ATPase subunit 1	+	+	25.92	7	6
Q53HV2	Chaperonin containing TCP1, subunit 7	+	+	25.99	7	6
Q53Y51	D-dopachrome tautomerase	+	n.d.	27.39	7	4
Q53Z07	NPC-A-16	+	+	39.52	10	4
Q562Z4	Actin-like protein	+	+	36.67	9	4
Q58FF6	Putative heat shock protein HSP 90-beta 4	+	+	46.80	13	5
Q597H1	Transformation-related protein 14	+	+	47.00	13	5
Q59EA2	Coronin isoform	+	+	47.02	13	3
Q59EF6	Calpain 2, large [catalytic] subunit variant	+	+	47.02	13	3
Q59ET0	Glucan , branching enzyme 1 variant	+	+	47.13	13	12
Q59F66	DEAD box polypeptide 17 isoform p82	+	+	47.24	13	10
Q59FD4	Hexokinase	+	+	26.92	7	6
Q59FF0	EBNA-2 co-activator	+	+	28.10	7	4
Q59FI4	Importin 4 variant	+	+	28.15	7	6
Q59FR8	Galectin	+	+	28.23	7	4
Q59G75	Isoleucyl-tRNA synthetase, cytoplasmic	+	+	26.72	7	6
Q59GB4	Dihydropyrimidinase-like 2	+	+	27.51	7	6
Q59GW5	Tripartite motif-containing 25	+	+	54.59	16	9
Q59GW6	Acetyl-CoA acetyltransferase, cytosolic	+	+	36.92	9	4
Q59GX2	Solute carrier family 2	+	+	60.77	20	7
Q59GY2	Ribosomal protein L4 variant	+	+	32.96	8	6
Q59H77	T-complex protein 1 subunit gamma	+	+	58.53	18	10
Q59HH3	Phosphoribosylglycinamide formyltransferase, phosphoribosylglycinamide synthetase	+	+	38.46	10	6
Q5JR94	40S ribosomal protein S8	+	+	40.58	11	3
Q5M7Z5	GRHPR protein	n.d.	+	28.65	7	3
Q5SU16	Beta 5-tubulin	+	+	28.66	7	6

Q5T0I0	Gelsolin	+	+	29.19	7	4
Q5T0R9	Adenylyl cyclase-associated protein	+	+	28.72	7	4
Q5T5C7	Serine--tRNA ligase, cytoplasmic	+	+	34.91	9	5
Q5T9B7	Adenylate kinase isoenzyme 1	+	+	46.36	13	6
Q5TB52	3'-phosphoadenosine 5'-phosphosulfate synthase 2	+	+	46.42	13	5
Q5TCI8	Prelamin-A/C	+	+	46.71	13	3
Q5U077	L-lactate dehydrogenase	+	+	46.72	13	4
Q5U5J2	CSNK2A1 protein	+	+	29.22	7	3
Q5VSQ6	Procollagen-proline, 2-oxoglutarate 4-dioxygenase	+	+	29.32	8	4
Q5VWC4	26S proteasome non-ATPase regulatory subunit 4	+	+	40.66	11	11
Q5VXV3	SET	+	+	59.50	19	3
Q65ZQ3	FBRNP	+	+	29.33	8	7
Q6DD88	Atlastin-3	+	+	30.08	8	7
Q6FGH5	RPS21 protein	+	+	34.40	9	4
Q6FHV6	ENO2 protein	+	+	30.10	8	7
Q6FHX6	FEN1 protein	+	+	30.15	8	6
Q6FI13	Histone H2A type 2-A	n.d.	+	29.52	8	7
Q6FIC5	Chloride intracellular channel protein	+	+	29.60	8	8
Q6IAW5	CALU protein	+	+	29.87	8	3
Q6IAX2	RPL21 protein	+	+	34.31	9	5
Q6IPH7	RPL14 protein	+	+	34.01	8	7
Q6IQ30	Polyadenylate-binding protein	+	+	44.88	12	12
Q6NZI2	Polymerase I and transcript release factor	+	+	45.00	12	3
Q6P2Q9	Pre-mRNA-processing-splicing factor 8	+	+	45.07	12	12
Q6YN16	Hydroxysteroid dehydrogenase-like protein 2	+	+	30.43	8	4
Q71U36	Tubulin alpha-1A chain	+	+	45.33	12	4
Q71UA4	Adenylosuccinate lyase	+	+	30.50	8	3
Q76LA1	CSTB protein	+	+	30.52	8	8
Q7Z4H8	KDEL motif-containing protein 2	+	+	28.33	7	2
Q86Y56	Dynein assembly factor 5	+	+	28.36	7	6
Q8N1G4	Leucine-rich repeat-containing protein 47	+	+	28.42	7	4
Q8N7G1	Purine nucleoside phosphorylase	+	+	52.63	15	10
Q8NBJ5	Procollagen galactosyltransferase 1	+	+	30.81	8	4
Q8NBS9	Thioredoxin domain-containing protein 5	+	+	71.60	29	10
Q8NC51	Plasminogen activator inhibitor 1 RNA-binding protein	+	+	38.73	10	8
Q8TCT9	Minor histocompatibility antigen H13	+	+	30.00	8	3
Q8TDN6	Ribosome biogenesis protein BRX1	n.d.	+	33.21	8	5
Q8TE01	DERP12	+	+	47.52	13	5
Q92499	ATP-dependent RNA helicase DDX1	+	+	47.53	13	6
Q92526	T-complex protein 1 subunit zeta-2	+	+	47.57	13	6
Q92688	Acidic leucine-rich nuclear phosphoprotein 32	+	+	47.77	13	5
Q92734	Protein TFG	+	+	52.38	15	7
Q92900	Regulator of nonsense transcripts 1	+	+	33.23	8	3
Q969H8	Myeloid-derived growth factor	+	+	30.85	8	5

Q969S3	Zinc finger protein 622	n.d.	+	66.36	24	3
Q96AG4	Leucine-rich repeat-containing protein 59	+	+	31.05	8	4
Q96C19	EF-hand domain-containing protein D2	+	+	51.24	15	15
Q96F07	Cytoplasmic FMR1-interacting protein 2	+	+	51.33	15	5
Q96G03	Phosphoglucomutase-2	+	+	51.37	15	8
Q96HE7	ERO1-like protein alpha	+	+	30.89	8	7
Q96KP4	Cytosolic non-specific dipeptidase	+	+	31.44	8	3
Q96QK1	Vacuolar protein sorting-associated protein 35	+	+	75.00	32	7
Q96T76	MMS19 nucleotide excision repair protein homolog	+	+	32.10	8	7
Q99426	Tubulin-folding cofactor B	n.d.	+	66.25	24	4
Q99497	Protein deglycase DJ	+	+	32.13	8	7
Q99536	Synaptic vesicle membrane protein VAT-1	+	+	57.69	18	4
Q99623	Prohibitin-2	+	+	57.69	18	2
Q9BQ39	ATP-dependent RNA helicase DDX50	n.d.	+	57.82	18	5
Q9BQG0	Myb-binding protein 1A	+	+	57.83	18	8
Q9BR76	Coronin-1B	+	+	58.02	18	2
Q9BRA2	Thioredoxin domain-containing protein 17	+	+	71.43	29	19
Q9BS26	Endoplasmic reticulum resident protein 44	+	+	32.27	8	3
Q9BUF5	Tubulin beta-6 chain	+	+	68.63	26	14
Q9BVA1	Tubulin beta-2B chain	n.d.	+	58.90	18	4
Q9BXP5	Serrate RNA effector molecule homolog	+	+	59.47	19	7
Q9BY44	Eukaryotic translation initiation factor 2A	+	+	51.94	15	4
Q9BZT4	PNAS-29	+	n.d.	52.01	15	7
Q9BZZ5	Apoptosis inhibitor 5	+	+	32.41	8	5
Q9H0U4	Ras-related protein Rab-1B	+	+	31.54	8	4
Q9H488	GDP-fucose protein O-fucosyltransferase 1	+	+	51.69	15	2
Q9HAV7	GrpE protein homolog 1, mitochondrial	+	+	51.81	15	6
Q9HBB3	60S ribosomal protein L6	+	+	51.89	15	11
Q9HDC9	Adipocyte plasma membrane-associated protein	n.d.	+	58.04	18	2
Q9NQC3	Reticulon-4	+	+	58.41	18	12
Q9NR30	Nucleolar RNA helicase 2	+	+	58.41	18	9
Q9NR45	Sialic acid synthase	+	+	58.43	18	4
Q9NSD9	Phenylalanine-tRNA ligase beta subunit	+	+	58.44	18	4
Q9NSE4	Isoleucine-tRNA ligase, mitochondrial	+	+	32.43	8	3
Q9NTJ3	Structural maintenance of chromosomes protein 4	+	+	63.87	23	3
Q9NYF8	Bcl-2-associated transcription factor 1	n.d.	+	32.52	8	5
Q9NZ23	Drug-sensitive protein 1	+	+	52.85	16	14
Q9NZM1	Myoferlin	n.d.	+	55.76	16	2
Q9NZN4	EH domain-containing protein 2	+	+	55.81	16	3
Q9P287	BRCA2 and CDKN1A-interacting protein	+	+	66.67	24	23
Q9UBF2	Coatomer subunit gamma-2	n.d.	+	66.67	25	11
Q9UHD8	Septin-9	+	+	66.67	25	9
Q9UHX1	Poly(U)-binding-splicing factor PUF60	+	+	52.63	15	13
Q9UKM9	RNA-binding protein Raly	+	+	57.44	17	4

Q9UMS4	Pre-mRNA-processing factor 19	+	+	52.87	16	7
Q9UQM3	Alpha-tubulin	+	+	53.00	16	8
Q9Y230	RuvB-like 2	+	+	55.67	16	4
Q9Y266	Nuclear migration protein nudC	+	+	55.42	16	6
Q9Y295	Developmentally-regulated GTP-binding protein 1	n.d.	+	53.06	16	12
Q9Y383	Putative RNA-binding protein Luc7-like 2	+	+	53.40	16	15
Q9Y3F4	Serine-threonine kinase receptor-associated protein	+	+	53.68	16	3
Q9Y3I0	tRNA-splicing ligase RtcB homolog	+	+	62.15	21	9
Q9Y3U8	60S ribosomal protein L36	+	+	56.07	16	6
Q9Y490	Talin-1	+	+	56.52	17	5
Q9Y4L1	Hypoxia up-regulated protein 1	+	+	56.68	17	6
Q9Y570	Protein phosphatase methylesterase 1	+	+	64.29	24	4
Q9Y5K6	CD2-associated protein	+	+	56.57	17	6
Q9Y678	Coatomer subunit gamma-1	+	+	64.70	24	24
Q9Y6E3	HSPC027	+	+	65.15	24	2
V9HW26	ATP synthase subunit alpha	+	+	56.37	17	10
V9HW31	ATP synthase subunit beta	+	+	53.85	16	3
V9HW62	Lactoylglutathione lyase	+	+	53.99	16	5
V9HW85	Phosphoglycerate kinase	+	+	54.44	16	6
V9HW88	Calreticulin	+	+	66.67	25	7
V9HWB8	Pyruvate kinase	+	+	66.87	25	3
V9HWE0	Annexin isoform	+	+	67.15	25	16
V9HWI3	Cathepsin D	+	+	67.26	25	5
V9HWJ1	Glutathione synthetase	+	+	67.43	25	19
X5DR09	General transcription factor IIi isoform A	+	+	67.81	25	16

Table S2.
The p-values and fold change (FC) for individual statistically significant proteins indicated in fibroblasts isolated from skin of psoriatic patients (n=5) and healthy people (n=5). Data analyzed using MetaboAnalyst 4.0 following log transformation.

Protein ID	FC	p-value	FDR
A0A024QYX3	0.24477	0.046604	0.11324
A0A024QZP7	0.46334	0.044295	0.11324
A0A024QZS4	5.9655	0.018749	0.092519
A0A024QZY1	0.43523	0.0043705	0.050529
A0A024R0E2	0.48716	0.035226	0.11105
A0A024R0E5	0.41587	0.0042131	0.050098
A0A024R0P9	0.21141	0.027652	0.10605
A0A024R1S8	0.21706	0.02545	0.10132
A0A024R5Z7	0.33353	0.0053433	0.053073
A0A024R6W0	0.39118	0.0035973	0.050098
A0A024R7B7	0.31135	0.0011627	0.032837
A0A024R814	0.48618	0.024937	0.10132
A0A024RA75	0.075029	0.0024	0.046655
A0A024RAD5	0.34661	0.043767	0.11322
A0A024RAI1	0.28738	6.70E-05	0.0063192
A0A024RAZ7	0.38313	0.0032728	0.047835
A0A024RBB7	0.44613	0.0042939	0.050529
A0A024RCN6	0.46686	0.032178	0.10976
A0A024RD93	0.41342	0.012117	0.079229
A0A024RDF6	0.43144	0.0041099	0.050098
A0A087X1U6	0.06669	0.0048141	0.050777
A0A087X1Z3	0.46825	0.035682	0.11105
A0A087X2I1	0.35271	0.039306	0.11215
A0A0A0MRI2	0.13418	0.010535	0.072142
A0A0A0MSE2	0.36228	0.0063235	0.057587
A0A0A0MTN0	0.47766	0.0135	0.082571
A0A0A0MTS2	0.46716	0.020131	0.092519
A0A0C4DG17	0.48838	0.024253	0.10132
A0A0C4DG89	0.30075	0.0050053	0.050777
A0A0D9SF53	0.48877	0.047412	0.11324
A0A0F7KYT8	0.48835	0.01557	0.087079
A0A0S2Z2Z6	0.32939	0.012608	0.080043
A0A0S2Z491	0.4125	0.0013777	0.034672
A0A0S2Z4A5	0.47597	0.032321	0.10976
A0A0S2Z5I7	0.31619	0.02809	0.10605
A2A3R6	0.31546	0.00017958	0.010429
A2RUM7	0.23352	0.034508	0.11048
A8K3S1	0.31718	0.027049	0.10605
A8K5M4	0.37092	0.026248	0.10345
B0QY89	0.40175	0.013624	0.083544
B2R5W3	0.058346	0.034487	0.11026
B3KS98	0.25419	0.023451	0.10132
B3KSH1	0.48722	0.012127	0.079229
B5BU01	0.13673	0.0094398	0.069308
B5BUB1	0.40065	0.0095981	0.069308
B7Z3K9	0.3489	0.004883	0.050777
B9VP24	0.22802	0.045147	0.11324
E7EPK1	0.20007	0.044158	0.11322
E9PAV3	0.47332	0.032244	0.10976
E9PB61	0.30221	0.0034213	0.050098
E9PRY8	0.42456	0.015378	0.086508
F4ZW62	0.40393	0.0097307	0.069308
F6WQW2	3.2108	0.0044945	0.050777
G3V4C1	0.44731	0.029999	0.1093
G5EA09	0.23878	0.045847	0.11324

H7C2I1	0.39667	0.022399	0.097362
J3KQE5	0.34389	3.42E-05	0.0036906
J9R021	0.45984	0.028844	0.10605
K7ELC2	0.17151	0.029311	0.107
L0R5C4	0.11942	0.0094865	0.069308
M0QXB4	0.23307	0.021259	0.094392
O00299	0.47892	0.010025	0.069308
O00487	0.35583	0.0088083	0.06786
O15143	0.46237	0.036297	0.11215
O15144	0.26929	0.0056939	0.053504
O15260	0.38344	0.00017749	0.010429
O15371	0.44306	0.013233	0.08138
O15397	0.046213	0.00060549	0.021769
O43143	0.43538	0.042383	0.11322
O43615	0.36882	0.038081	0.11215
O60506	0.49538	0.043664	0.11322
O60664	0.27858	0.0030371	0.047696
O75390	0.49382	0.036216	0.11215
O75643	0.4107	0.039717	0.11322
O76003	85.775	0.003285	0.048476
O95678	0.31671	0.0308	0.10976
O96019	0.3825	0.036817	0.11215
P00387	0.19388	0.015529	0.087079
P00558	0.4978	0.018027	0.091303
P01375	5.8637	0.01179	0.078003
P04075	0.3261	0.00057171	0.021718
P04083	0.41571	0.0082683	0.065026
P04350	0.19219	0.034791	0.11105
P04406	0.28052	0.00057531	0.021718
P05023	0.47479	0.015559	0.087079
P05109	34.814	0.00194297	0.046655
P05198	0.47946	0.021536	0.096193
P05556	0.49842	0.039135	0.11215
P06702	20.057	0.041635	0.11322
P07205	0.37503	0.0028745	0.047696
P07384	0.095587	0.00053138	0.021718
P07954	0.34836	0.00092517	0.02794
P08134	0.093412	0.0018866	0.046655
P08263	323.49	1.35E-12	1.02E-09
P08727	0.40708	0.03589	0.11215
P0CG39	0.49055	0.047643	0.11324
P10644	0.21155	0.042012	0.11322
P11177	0.48573	0.018719	0.092519
P11216	0.39668	0.025631	0.10175
P11908	0.22023	0.018235	0.092519
P12004	0.38022	0.0033769	0.048499
P12814	0.48059	0.025453	0.10173
P13639	0.4979	0.043784	0.11322
P13667	0.48754	0.029195	0.107
P14866	0.35166	0.0073587	0.063472
P15880	0.45051	0.021142	0.093364
P17174	0.48399	0.0027672	0.047696
P17931	0.052577	0.0023803	0.046655
P17980	0.48681	0.019029	0.092519
P18085	0.17417	0.014193	0.083625
P19012	0.22143	0.022947	0.099143
P20618	0.18969	0.034075	0.11002
P21796	0.486	0.026399	0.10345
P22626	0.32892	0.0018086	0.041893
P23246	0.49193	0.046045	0.11324
P23381	0.47529	0.032013	0.10976
P23396	0.47139	0.0041163	0.050098

P23528	0.49651	0.0003939	0.018587
P24752	0.28916	0.0018848	0.046655
P25786	0.47071	0.022593	0.097362
P25788	0.47356	0.026503	0.10577
P26368	0.42583	0.0024452	0.046926
P26583	18.464	0.0015314	0.041893
P27348	4.5382	0.00083014	0.026115
P27695	4.7399	0.0012087	0.032837
P30040	2.0903	0.01956	0.092519
P30048	2.0545	0.0051402	0.051066
P30101	2.4322	0.008998	0.067935
P30153	2.1034	0.021202	0.094392
P31947	3.5845	0.00015577	0.010429
P35222	0.026502	0.0006398	0.021957
P35527	208.9	6.33E-09	2.39E-06
P35908	2.7238	0.039533	0.11321
P35998	2.111	0.016644	0.087079
P37837	2.5242	0.015039	0.085977
P38646	2.2701	0.0078245	0.063472
P40925	2.0994	0.019167	0.092519
P40926	2.5394	0.0039807	0.050098
P41091	2.5122	0.022861	0.098472
P41250	2.1028	0.025791	0.10175
P43686	2.1325	0.012935	0.080043
P46934	3.1308	0.030719	0.1093
P49736	2.1314	0.039486	0.11321
P50502	2.3068	0.033903	0.10976
P51991	2.278	0.012616	0.080043
P52209	2.1597	0.029365	0.107
P52566	3.9813	0.023175	0.099518
P53618	2.0441	0.043055	0.11322
P53621	2.4138	0.039799	0.11322
P53992	190.53	5.11E-07	9.64E-05
P54886	3.5108	0.029791	0.10805
P55060	2.3329	0.025846	0.10345
P55263	3.696	0.0030036	0.047696
P61160	4.5194	0.00082719	0.026115
P61163	9.5901	0.0079626	0.063472
P61221	2.2901	0.016958	0.088099
P61970	5.3533	0.017914	0.091303
P62244	3.54	0.031948	0.10976
P62258	2.2215	0.0069285	0.058955
P62917	2.3362	0.010235	0.07074
P62942	2.4915	0.049772	0.11483
P63104	2.7217	0.0012906	0.033601
P67775	3.0055	0.0024498	0.047696
P68371	3.165	0.0032109	0.047696
P80723	2.9699	0.0047416	0.050777
Q00653	2.3012	0.0014994	0.037296
Q05655	0.0078133	0.00029113	0.0157
Q07960	11.295	0.0042601	0.050098
Q08AJ9	4.9954	0.017481	0.09106
Q13148	2.3756	0.029094	0.10605
Q13151	2.1797	0.01587	0.087079
Q13185	0.45156	0.048333	0.11439
Q13200	2.0722	0.033436	0.10976
Q13501	2.4037	0.0096389	0.069308
Q14103	2.6621	0.0028025	0.047696
Q14247	2.7953	0.0050364	0.050777
Q14444	3.6107	0.029581	0.10805
Q14566	2.3614	0.014462	0.085727
Q14683	2.5378	0.044382	0.11324

Q14974	2.5333	0.012173	0.079229
Q15019	2.6002	0.010415	0.07155
Q15056	3.08	0.0035313	0.050098
Q15181	2.1721	0.018323	0.092519
Q15393	2.6755	0.0045509	0.050777
Q15417	7.9389	0.0072826	0.06039
Q16236	86.626	0.0032363	0.047696
Q16531	2.0641	0.033263	0.10976
Q16629	2.1207	0.031864	0.10976
Q16881	2.2892	0.0054519	0.053504
Q2NKY5	3.2964	8.58E-05	0.0072014
Q32P28	2.9215	0.02052	0.093293
Q3ZCR3	6.3223	0.018389	0.092519
Q4LE58	2.1669	0.017051	0.090346
Q53HB3	2.3583	0.0047944	0.050777
Q58FF6	9.6655	0.018666	0.092519
Q597H1	3.6412	0.0024718	0.047696
Q59EF6	2.3785	0.0051351	0.051066
Q59F66	3.8737	0.028476	0.10605
Q59FD4	2.657	0.0053079	0.051453
Q59FF0	2.2012	0.034555	0.11105
Q59GW6	2.8542	0.0043794	0.050777
Q5M7Z5	5.892	0.011675	0.077542
Q5SU16	2.1597	0.0070278	0.06039
Q5U077	2.4306	0.0086701	0.067484
Q5U5J2	2.8239	0.003157	0.047696
Q5VSQ6	2.4494	0.022836	0.098472
Q5VWC4	3.3766	1.68E-07	4.24E-05
Q6FHV6	2.826	0.023446	0.10132
Q6FHX6	2.0131	0.037109	0.11215
Q6FIC5	5.235	0.0012178	0.032837
Q6IQ30	2.1123	0.041164	0.11322
Q8N7G1	2.1171	0.0041837	0.050098
Q8NBJ5	4.0804	0.045355	0.11324
Q8NC51	2.0832	0.00037859	0.018587
Q8TDN6	6.0694	0.011197	0.072307
Q92499	2.7391	0.0066359	0.058955
Q92526	16.626	0.0032363	0.047835
Q96AG4	4.6136	0.019628	0.092519
Q96C19	2.0739	0.047692	0.11324
Q96G03	3.039	0.017933	0.091303
Q96HE7	2.3422	0.014343	0.085727
Q99426	4.6584	0.022739	0.098472
Q99757	2.4125	0.0058819	0.056123
Q9BQ39	6.334	0.018246	0.092519
Q9BR76	2.3683	0.016839	0.087079
Q9BUF5	4.0255	0.00013313	0.010051
Q9BVA1	273.56	7.12E-06	0.0010758
Q9H488	5.1637	0.03179	0.10976
Q9HBB3	2.2956	0.0065986	0.057587
Q9HDC9	2.747	0.034662	0.11105
Q9NR45	2.7511	0.0025483	0.047696
Q9NYF8	9.2195	0.0069855	0.058955
Q9NZ23	2.0039	0.024161	0.10132
Q9UKM9	2.057	0.041635	0.11322
Q9UQM3	5.9969	1.13E-05	0.0014277
Q9Y383	2.6409	0.011503	0.07616
Q9Y3F4	3.4814	0.0094297	0.067935
Q9Y4L1	2.3012	0.014994	0.085945
V9HW88	2.1113	0.002437	0.046655
V9HWB8	2.5345	0.0038019	0.050098
V9HWE0	2.3357	0.016394	0.087079

V9HWI3	2.4421	0.0058819	0.056167
